# A body composition model with multiple storage compartments for polar bears (*Ursus maritimus*)

**DOI:** 10.1093/conphys/coad043

**Published:** 2023-06-20

**Authors:** Stephanie R Penk, Pranav Sadana, Louise C Archer, Anthony M Pagano, Marc R L Cattet, Nicholas J Lunn, Gregory W Thiemann, Péter K Molnár

**Affiliations:** Laboratory of Quantitative Global Change Ecology, Department of Biological Sciences, University of Toronto Scarborough, 1265 Military Trail, Scarborough, Ontario M1C 1A4, Canada; Department of Ecology and Evolutionary Biology, University of Toronto, 25 Willcocks Street, Toronto, Ontario M5S 3B2 Canada; Laboratory of Quantitative Global Change Ecology, Department of Biological Sciences, University of Toronto Scarborough, 1265 Military Trail, Scarborough, Ontario M1C 1A4, Canada; Department of Biology, University of Winnipeg, 515 Portage Ave, Winnipeg, Manitoba R3B 2E9, Canada; Laboratory of Quantitative Global Change Ecology, Department of Biological Sciences, University of Toronto Scarborough, 1265 Military Trail, Scarborough, Ontario M1C 1A4, Canada; U.S. Geological Survey, Alaska Science Center, 4210 University Dr., Anchorage, AK 99508 USA; Fish and Wildlife Branch, Department of Environment, Government of Yukon, 10 Burns Road, Whitehorse, Yukon Y1A 4Y9, Canada; Wildlife Research Division, Science and Technology Branch, Environment Canada and Climate Change Canada, 11455 Saskatchewan Dr., Edmonton, Alberta T6G 2E9, Canada; Faculty of Environmental and Urban Change, York University, 4700 Keele St., Toronto, Ontario M3J 1P3, Canada; Laboratory of Quantitative Global Change Ecology, Department of Biological Sciences, University of Toronto Scarborough, 1265 Military Trail, Scarborough, Ontario M1C 1A4, Canada; Department of Ecology and Evolutionary Biology, University of Toronto, 25 Willcocks Street, Toronto, Ontario M5S 3B2 Canada

## Abstract

Climate warming is rapidly altering Arctic ecosystems. Polar bears (*Ursus maritimus*) need sea ice as a platform from which to hunt seals, but increased sea-ice loss is lengthening periods when bears are without access to primary hunting habitat. During periods of food scarcity, survival depends on the energy that a bear has stored in body reserves, termed storage energy, making this a key metric in predictive models assessing climate change impacts on polar bears. Here, we developed a body composition model for polar bears that estimates storage energy while accounting for changes in storage tissue composition. We used data of dissected polar bears (*n =* 31) to link routinely collected field measures of total body mass and straight-line body length to the body composition of individual bears, described in terms of structural mass and two storage compartments, adipose and muscle. We then estimated the masses of metabolizable proteins and lipids within these storage compartments, giving total storage energy. We tested this multi-storage model by using it to predict changes in the lipid stores from an independent dataset of wild polar bears (*n* = 36) that were recaptured 8–200 days later. Using length and mass measurements, our model successfully predicted direct measurements of lipid changes via isotopic dilutions (root mean squared error of 14.5 kg). Separating storage into two compartments, and allowing the molecular composition of storage to vary, provides new avenues for quantifying energy stores of individuals across their life cycle. The multi-storage body composition model thus provides a basis for further exploring energetic costs of physiological processes that contribute to individual survival and reproductive success. Given bioenergetic models are increasingly used as a tool to predict individual fitness and population dynamics, our approach for estimating individual energy stores could be applicable to a wide range of species.

## Teaser

We related straight-line body length and total body mass of polar bears to their body composition and associated storage energy using a dataset of dissected polar bears. Individual survival and reproduction can subsequently be predicted by estimating polar bear energy demands relative to their estimated energy stores.

## Introduction

Rapid environmental changes characteristic of the Anthropocene are altering habitats worldwide, including shifts in resource availability caused by rapid changes in ocean/atmospheric temperatures and precipitation (Walther *et al.*, 2002; Segan *et al.*, 2016; Masson-Delmotte *et al.*, 2021; Habibullah *et al.*, 2022). The Arctic warmed at more than twice the global rate between 1970 and 2000, and upwards of four times the global average over the past two decades (Masson-Delmotte *et al.*, 2021; Chylek *et al.*, 2022). Warming has led to declines in Arctic sea ice, which is used by polar bears (*Ursus maritimus*) as a platform to hunt their primary food source—seals (Stirling and Øritsland, 1995; Thiemann *et al.*, 2006; Pilfold *et al.*, 2012). In regions where sea ice cover is seasonal, polar bears are already undergoing shifts in food availability (Thiemann *et al.*, 2008; Stirling, 2012; Pilfold *et al.*, 2016) due to warmer temperatures lengthening the summer ice-free periods when polar bears do not have access to seals and decreasing the amount of time that bears are able to spend hunting on the sea ice (Hunter *et al.*, 2010; Rode *et al.*, 2012; Stirling, 2012; Stirling and Derocher, 2012). Climate projections suggest that Arctic warming and associated changes in sea ice availability will continue and accelerate (Masson-Delmotte *et al.*, 2021). A key challenge for conservation managers is to assess how species, such as polar bears, will respond to yet-unobserved environmental conditions, for example, to determine conservation priorities or to assess sustainable harvest quotas (Molnár *et al.*, 2010; Keys *et al.*, 2019). Individual physiology—in particular, measures of energy stores—can provide a mechanistic link between fitness and resource availability, facilitating prediction and population inference for future environmental conditions (Nisbet *et al.*, 2000; Kooijman, 2010; Tomlinson *et al.*, 2014; Molnár *et al.*, 2020).

Energy stores are often inferred from an individual’s body condition, whereby ‘good’ body condition suggests large energy stores for a given size or age (Speakman, 2001). A quantitative index of body condition typically consists of total body mass and a morphometric correlate of structural size (Kotiaho, 1999; Cattet *et al.*, 2002; Peig and Green, 2009) (e.g. height in humans, fork length in fish, tarsus length in birds). Structural size is an indicator of structural mass, i.e. the tissue mass that is assumed to be unavailable to metabolize as energy even under starvation—hereafter referred to as ‘structure’ or ‘structural mass’ (Kooijman, 2010). On the other hand, tissue mass that can be regularly metabolized as an energy source is termed ‘storage mass’, and the energy contained within is referred to as ‘storage energy’ (Kooijman, 2010). Although body condition indices are useful indicators of individual health and fitness in wildlife, estimating an individual’s storage energy can be valuable for many species, particularly for those that experience large fluctuations in food availability—when storage energy may limit ability to complete key life-history functions, e.g. for fish (Sogard and Olla, 2000; Pangle *et al.*, 2011), mammals (Humphries *et al.*, 2003; Vucetich and Peterson, 2004; de Roos *et al.*, 2009; Johnsen *et al.*, 2017), reptiles (Zani *et al.*, 2012) and insects (Sinclair, 2015).

To accurately estimate an individual’s storage energy, knowledge of body composition—the relative proportions of body tissues and their molecular makeup—is required because different tissue types vary in their chemical composition. For example, the energy density provided by lipids stored in body fat (39.3 MJ/kg) is more than twice that of carbohydrates (15.7 MJ/kg) or protein (18.4 MJ/kg) (Elia and Cummings, 2007), meaning that tissues rich in lipids (e.g. adipose) provide significantly higher energy than those with a relatively high proportion of protein (e.g. muscle) (Blaxter, 1989). Direct determination of the potential energy available to an individual is possible via chemical extraction of the molecular components in storage—water, lipids, protein, ash—but requires sacrificing the animal (Farley and Robbins, 1994; Speakman, 2001).

For polar bears, various non-destructive methods have been developed to describe body condition, including a subjective fatness index (Stirling *et al.*, 1989, 2008), lipid content of adipose biopsies (Thiemann *et al.*, 2006; Stirling *et al.*, 2008; McKinney *et al.*, 2014; Sciullo *et al.*, 2016) and morphometric indices that use a combination of body mass and length (e.g. Cattet *et al.*, 2002). However, although useful as indicators of energetic status, none of these indices provide a quantitative estimate of storage energy.

Isotopic dilution and bioelectrical impendence analyses are non-destructive methods used to quantify individual body composition in terms of fat mass (all polar and nonpolar lipids regardless of their function, henceforth referred to as ‘total lipid mass’), and fat-free mass (all non-lipid molecular components, i.e. protein, minerals, water, henceforth referred to as ‘lean body mass’) (Farley and Robbins, 1994; Atkinson *et al.*, 1996; Hilderbrand *et al.*, 1998). Given the major role lipids play in surviving periods of low resource availability (Welte and Gould, 2017a; Cockcroft, 2021), total lipid mass is commonly used as a metric of energy stores to facilitate estimates of fasting lengths and time to starvation (Robbins *et al.*, 2012). Lipids may also be structural (e.g. footpads) (Pond *et al.*, 1992; Cockcroft, 2021), however, and thus unavailable as an energy source, while some protein included in lean body mass may be metabolized as energy (e.g. the collagen in the extracellular matrix of adipose tissue (Ramsay *et al.*, 1992)). As the depletion of protein stores and the associated breakdown of critical structural components to compensate is often the primary cause of death in starving individuals (Caloin, 2004; Robbins *et al.*, 2012), including metabolizable protein as an energy source may be important when predicting survival. Although fasting limits for polar bears have been estimated using body composition determined via isotopic dilution (Robbins *et al.*, 2012), estimates of the proportion of protein and lipid that are available as energy sources may improve the accuracy of predicted fasting lengths.

An alternative approach, the storage energy body composition model (Molnár *et al.*, 2009), estimates body composition in terms of structural mass and storage mass based on an individual’s straight-line body length and total body mass. The model assumes both isomorphy (i.e. structural shape is constant with ageing) and strong homeostasis, whereby the respective chemical compositions of structural and storage masses do not change over time (i.e. the energy contained within a unit of storage mass is invariant even as the total body mass of the bear changes) (Kooijman, 2010). However, the assumption of strong homeostasis may not always hold given the extreme cycles of feeding and fasting in polar bears. For example, although bears appear to preferentially store and deplete lipid-rich adipose tissue as an energy source (Ramsay *et al.*, 1991; Pond *et al.*, 1992; Atkinson and Ramsay, 1995; Atkinson *et al.*, 1996), muscular atrophy (Whiteman *et al.*, 2017) and loss of lean body mass have also been observed in fasting individuals, especially those in poor condition (i.e. lower percentages of adipose tissue) (Ramsay *et al.*, 1991; Pond *et al.*, 1992; Atkinson and Ramsay, 1995; Atkinson *et al.*, 1996). These observations suggest that both adipose and muscle tissue comprise storage mass in polar bears but may be broken down at different rates depending on individual body condition. Adipose and muscle tissue have different macronutrient profiles, with adipose tending to contain relatively more energy-rich lipids than muscle tissue (Supplementary Table S2), suggesting that the energy contained in a unit of storage is not invariant but instead depends on the relative amounts of adipose and muscle in the storage mass.

Molecular analyses of storage tissues suggest that further variability in storage composition, and thus storage energy, may occur due to variation in the proportion of lipid and protein in both adipose tissue (45–75% lipid (Supplementary Fig. S3, Thiemann *et al.*, 2006), approximately 5–50% protein Supplementary Fig. S3) and muscle tissue (0.3–6.8% lipid (Supplementary Fig. S4), 32–62% protein (Whiteman *et al.*, 2017)). Changes in the volume and number of adipocytes—the cells that comprise adipose tissue—may underlie such changes in storage composition. When large lipid droplets within adipocytes are metabolized for energy (Ramsay *et al.*, 1992), the proportion of lipid in adipose tissue may decrease, while the proportions of the other molecular components (water, protein, minerals) increase (Pond *et al.*, 1992; Ramsay *et al.*, 1992; Birsoy *et al.*, 2013). Furthermore, activity levels can influence the molecular composition of muscle tissue (Whiteman *et al.*, 2017). Longitudinal variation in storage composition may thus occur within the same individual, suggesting that a single storage compartment assuming strong homeostasis may not fully reflect the storage energy of polar bears.

Here, we used body composition data from dissected polar bears (Cattet *et al.*, 2002) to develop a multi-storage body composition model (hereafter, the ‘multi-storage model’), which separates storage mass into two separate storage compartments, adipose and muscle. We then developed predictive relationships for lipid and protein content in each storage compartment, allowing the energy available in a unit of storage energy to vary with body condition and enabling the estimation of total storage energy for polar bears across a range of body lengths and masses, for both sexes and for all age classes. We tested the model by comparing predictions of lipid mass from the multi-storage model to estimates of lipid mass from isotopic dilution, and to predictions from a single-storage body composition model (hereafter, the ‘single-storage model’) (Molnár *et al.*, 2009).

## Methods

### Study systems and datasets

We used three distinct types of physiological data obtained from wild polar bears to develop the multi-storage body composition model: (i) mass and composition of different tissue types from harvested polar bears that were fully dissected (Cattet, 1988), hereafter ‘dissection data’; (ii) lipid content in adipose tissue biopsies from wild-caught polar bears (Sciullo *et al.*, 2016), hereafter ‘biopsy data’; and (iii) isotopic dilution measurements of total lipid mass from wild-caught bears (Arnould and Ramsay, 1994; Atkinson and Ramsay, 1995), hereafter ‘isotopic data’. The dissection data and biopsy data were used to develop and train the model, while the isotopic data were used to test the fitted model.

The dissection data were collected from polar bears harvested in the Foxe Basin (FB; *n* = 6), Western Hudson Bay (WH; *n* = 22) and Lancaster Sound (LS; *n* = 3) subpopulations between October 1985 and December 1986 (Cattet, 1988). The straight-line body length (i.e. the distance above the body contour from the tip of the nose to the end of the last tail vertebra when the spine is straightened), sex and age, of each bear was recorded, and the total body mass was determined by summing dissection components (Cattet, 1988). The age of the previously unmarked bears older than one year was determined by the extraction of a single, vestigial premolar during handling and subsequent counts of cementum annuli (Calvert and Ramsay, 1995). The age of cubs-of-the-year (COY, ~ 9 months old in autumn) and yearling cubs (~21 months old in autumn) was determined based on body size and dentition (Supplementary Table S1). Ages were modified where recorded total mass and straight-line body length fell outside the age-specific range for each measurement given by 45 years of data collected from WH (Supplementary Table S1). In this study, we defined individuals as adults based on the average age when 97% of the subpopulation’s asymptotic straight-line body length is reached and, thus, structural growth is largely complete (Derocher and Stirling, 1998; Rode *et al.*, 2010, 2020). Individuals were harvested across age groups as follows: adult females (4 years and older; *n* = 7), adult males (6 years and older; *n* = 8), subadult females (2–3 years inclusive; *n* = 3), subadult males (2–5 years inclusive; *n* = 9), yearlings (1 year olds, *n* = 3) and cubs-of-the-year (<1 year; *n* = 1) (Cattet, 1988). Tissues from each bear were separated into hide, skeletal muscle (henceforth ‘muscle’), adipose, viscera and all bones, including the brain and spinal cord contained within the cranium and spinal column (henceforth ‘bones’) (Cattet, 1988). All smooth and cardiac muscle tissues were included with visceral tissue where they serve a structural function. The chemical composition of each tissue type was determined in a subset of dissected polar bears (*n* = 5), with individuals chosen to represent a range of mass and body conditions (Supplementary Table S1, S2). Each sample for a given tissue type was composed of homogenized tissue taken from all major deposits of that tissue on the body (e.g. the muscle sample was taken from a homogenate of all major muscle depots) (Cattet, 1988). The proportions of water, ash, protein and the neutral lipid content –a non-polar class of lipids that includes triglycerides and is the main form of energy storage in mammals (Welte and Gould, 2017b)– within each homogenate sample were determined using standard laboratory assays (Cattet, 1988). Additional chemical analyses were performed to determine the proportions of water, lipid and combined ash and protein found in specific muscle depots (biceps femoris, *n* = 29; Supplementary Fig. S4) and specific adipose depots (subcutaneous, *n* = 25; Supplementary Fig. S3). See Cattet *et al.* (2001) for a detailed description of the chemical composition analysis.

Biopsy data were collected from adipose tissue of 140 polar bears from the Western Hudson Bay subpopulation in September 2007–2013 (Sciullo *et al.*, 2016). The samples included cubs-of-the-year (*n* = 11), yearlings (*n* = 5), subadult females (*n* = 12), subadult males (*n* = 13), adult females (*n* = 38) and adult males (*n* = 61). Subcutaneous adipose tissue was biopsied from each individual and analyzed for lipid content (i.e. both nonpolar and polar lipids; Supplementary Fig. S5) as a percentage of adipose (Sciullo *et al.*, 2016). Sex, age and straight-line body length were collected at each capture as well as total body mass measured with an electronic load cell suspended from an aluminum tripod.

The isotopic data contained repeated estimates for whole-body lipids of 36 bears from Western Hudson Bay (*n* = 32) and the Southern Beaufort Sea (*n* = 4), measured by isotopic dilution. See the **Model Testing** section below for a detailed description of the isotopic data.

### The multi-storage polar bear body composition model

**Figure 1:**
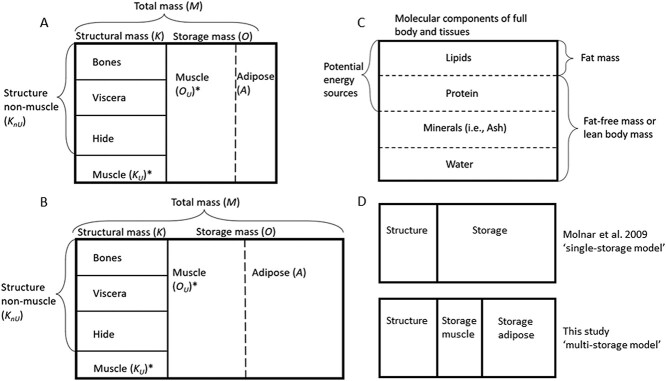
Schematic representation of the polar bear multi-storage body composition model, showing the tissue components of structure and storage compartments for a hypothetical adult polar bear in (A) relatively poorer and (B) higher body condition. Within our modelling framework, the variable ‘structure non-muscle’ (*K_nU_*) is considered the amalgamation of bone, viscera and hide. Total muscle is further divided into structural muscle (*K_U_*) and storage muscle (*O_U_*). The relative proportion of tissues within the structural compartment are assumed to be in strong homeostasis while those in the storage compartment are allowed to change based on body condition. The underlying molecular composition of tissues is shown in (C). Tissues are comprised of lipids, proteins, minerals (i.e. ash) and water, with the proportional composition varying based on hydration, body condition and tissue type. The sources of energy within tissues are highlighted, as well as the components that make up the fat-free mass (i.e. lean body mass) and fat mass that are typically estimated via isotopic dilution studies. Panel (D) shows a conceptual comparison between a single-storage model (Molnár *et al.*, 2009) and our multi-storage model developed here. The single-storage model contains two model compartments (structure and storage), while the multi-storage model further divides storage into two sub-compartments, storage adipose and storage muscle. The energy density of storage mass within the single-storage model is constant within sex/age classes. In the multi-storage model, the ratio of storage muscle to adipose and the proportion of lipid within adipose both vary with body condition, meaning the energy density of total storage mass can change within an individual (e.g. the bear in panel B would have higher storage energy than in panel A). Variables indicated with a star (*) were estimated in this study. Note that the potential energy sources in (C) do not distinguish between metabolizable versus structural molecules (i.e. lipids and protein both contain structural and storage molecules).

We first partitioned total body mass into a structural mass compartment (*K*) and storage mass (*O*), and next partitioned storage mass into two sub-compartments: storage adipose mass (A) and storage muscle mass (*O_U_*) (Fig. 1):

$$\begin{align*}Total\ Mass\ (M)= Structural\ Mass\ (K)+ Storage\ Mass\ (O) \end{align*}$$

$$\begin{align*} Storage\ Mass\ (O)&= Storage\ Muscle\ Mass\ \left({O}_U\right) \\ &+ \ Adipose\ Mass\ (A) \end{align*}$$

We then developed mathematical equations that predict a polar bear’s total structural mass, total storage mass and the proportions of total storage mass that comprise the storage muscle compartment and the storage adipose compartment, respectively, from a bear’s straight-line body length and total body mass (Box 1). We parameterized all equations by fitting the models to the dissection data. A Bayesian analysis framework with Hamiltonian Monte Carlo sampling was adopted to fit all models using STAN version 2.27.0 via the ‘*rstan*’ package in R version 4.10 (Stan Development Team, 2020).

**Box 1:**
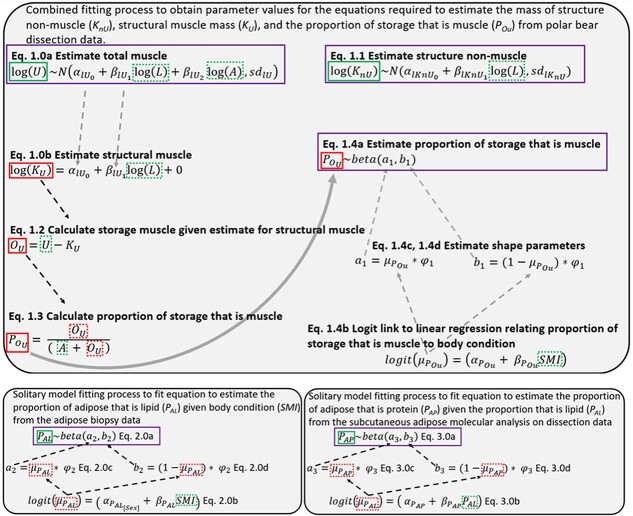
**The process of fitting the multi-storage body composition model components is shown for each step of the Bayesian model fitting framework.** The combined fitting process (top panel) uses the latent variable (i.e. unobservable variable that must be inferred through a model) of structural muscle mass (*K_U_*)—estimated from the fitted logged linear regression of the observed, measured mass of total muscle (*U*) as predicted by the measured variables of straight-line body length (*L*) and mass of adipose tissue (*A*) (Eqn. 1.0a, 1.0b)—to estimate the proportion of storage that is muscle (${P}_{O_U}$) (Eqn. 1.2, 1.3). A beta regression is used to simultaneously predict the estimated proportion of storage that is muscle from scaled mass index (*SMI*), a measure of body condition, (Eqn. 1.4a–d) so that the fitted equation 1.4b can predict the proportion of storage that is muscle from scaled mass index alone. Beta regressions to predict the proportion of adipose that is lipid (*P_AL_*) given the scaled mass index (bottom left) and the proportion of adipose that is protein (*P_AP_*) given proportion that is lipid (bottom right) were fitted separately. Equations which are fitted to data are outlined in purple. Measured outcome variables, which are observable and determined from existing datasets, used to fit equations are outlined in solid green squares while estimated latent variables are outlined in solid red squares. Measured predictor variables are outlined in dotted green squares, while estimated latent variables used as predictors are outlined in dotted red squares. Parameters which are estimated are not outlined.

Tissues from dissected polar bears were first assigned to one of the three compartments based on their function. All bones, hide and viscera (henceforth referred to as ‘structure non-muscle’) were assigned to the structural compartment, while all adipose tissue was assigned to the storage adipose compartment. Total muscle mass (*U*), which may include both structural (*K_U_*) and storage (*O_U_*) muscle (Cattet *et al.*, 2002), was separated into the structural and storage compartments by fitting Eqn. 1.0a, which estimates observed total muscle mass (*U*) as a function of straight-line body length (*L)* and adipose mass (*A)*:

\begin{align*} &\log (U)\sim N\left({\alpha}_{l{U}_0}+{\beta}_{lU_1}\log (L)+{\beta}_{lU_2}\log (A),{sd}_{lU}\right)\\& \textbf{Equation 1.0a}\ \text{Regression to fit total logged muscle.}\end{align*}

allowing total muscle to be predicted once fit. The mass of structural muscle associated with straight-line body length, our predictor of structure, can then be estimated from the fitted Eqn. 1.0a when the mass of adipose tissue, the predictor associated with storage muscle, is set to zero (Eqn. 1.0b).

\begin{align*} &\log \left({K}_U\right)={\alpha}_{lU_0}+{\beta}_{lU_1}\log (L)+0\\ &\textbf{Equation 1.0b}\ \text{Estimate logged structural muscle.} \end{align*}

To allow prediction of structure non-muscle (*K_nU_*), we fit the following log-linear equation, which estimates observed mass of structure non-muscle (*K_nU_*) from straight-line body length (*L*):

**Equation 1.1** Regression to fit logged structure non-muscle. \begin{align*} \log \left({K}_{nU}\right)\sim N\left({\alpha}_{lK{ nU}_0}+{\beta}_{lK{ nU}_1}\log (L),{sd}_{l{K}_{nU}}\right) \end{align*}

Structure non-muscle and structural muscle sums to give total structural mass (*K*), which in turn yields storage mass (*O*) as the difference between total body mass (*M*) and structural mass, from measured straight-line body length and total body mass alone (Fig. 1, Box 1, see Supplementary Materials 1.1).

$$\begin{align*} Structural\ Mass\ (K)&= Structural\ Muscle\ \left({K}_U\right)\\&+ Structure\ non-muscle\ \left({K}_{nU}\right) \end{align*}$$

$$\begin{align*} Storage\ Mass\ (O)= Total\ Mass\ (M)\hbox{---} Structural\ Mass\ (K) \end{align*}$$

Storage muscle (*O_U_*) is estimated as the difference between the observed mass of total muscle (*U*) and the estimated mass of structural muscle (*K_U_*; Eqn. 1.2). To estimate the proportion of storage that is muscle (${P}_{O_U}$) from the dissection data, we divided the estimated mass of storage muscle (*O_U_*) by the total mass of storage, given as the sum of the observed mass of adipose tissue (*A*) and predicted mass of storage muscle (Eqn. 1.3, see Supplementary Materials 1.1).

**Equation 1.2** Estimate storage muscle given Eqn. 1.0b. \begin{align*} {O}_U=U-\mathit{\exp}\left({\alpha}_{lU_0}+{\beta}_{l{U}_1}\log (L)\right) \end{align*}

\begin{align*} & \quad \quad \quad{P}_{O_U}=\frac{O_U}{\left(A+{O}_U\right)} \\ & \textbf{Equation 1.3}\ \textrm{ Estimate proportion of} \\ &{\text{ storage that is muscle given Eqn. 1.2.}} \end{align*}

Within the same fitting process (henceforth the ‘combined fitting process’), we developed a beta regression model that, once fit, predicts the proportion of storage that is muscle (${P}_{O_U}$) (as estimated by our model equations 1.0–1.3, Supplementary Materials 1.2) from an indicator of individual body condition known as the scaled mass index (SMI; Peig and Green, 2009), developed here for polar bears (see Supplementary Materials 2.0) (Eqn. 1.4a-d, Box 1 top panel). The use of SMI decreases the impact from measurement errors associated with straight-line body length (Peig and Green, 2009).

\begin{align*}& \quad\quad\quad\quad\quad{P}_{O_U}\sim beta\left({a}_1,{b}_1\right) \\& \textbf{Equation 1.4a}\ \textrm{ Beta regression to fit proportion}\\ &\textrm{of storage that is muscle} \end{align*}

\begin{align*} & \quad\quad\quad\quad\quad logit\left({\mu}_{P_{Ou}}\right)=\left({\alpha}_{P_{Ou}}+{\beta}_{P_{Ou}} SMI\right)\\& \textbf{Equation 1.4b} \textrm{ Logit link to linear regression} \\& \textrm{relating proportion of storage that is muscle to body condition} \end{align*}

**Equation 1.4c** First shape parameter for beta regression. \begin{align*} {a}_1={\mu}_{P_{Ou}}\ast{\varphi}_1 \end{align*}

**Equation 1.4d** Second shape parameter for beta regression. \begin{align*} {b}_1=\left(1-{\mu}_{P_{Ou}}\right)\ast{\varphi}_1 \end{align*}

We also fit a multi-level version of the model for storage muscle, where the intercept and slope in equation 1.4b (Eqn. S1, S2) was allowed to vary by sex to test for potential sex-related differences in the proportion of muscle in storage mass (see Supplementary Materials 1.3.1).

To estimate how adipose lipid content varies with body condition, we developed a multi-level beta regression to predict the proportion of adipose that is lipid (*P_AL_*) given the sex and body condition (*SMI*) of an individual (Eqn. 2.0a-d), which was fit using the biopsy data (Supplementary Table S4, Supplementary Fig. S5, Box 1 top and bottom left panels, see Supplementary Materials 1.4 and 3.2).

\begin{align*}&\quad\quad\quad\quad\quad\quad{P}_{AL}\sim beta\left({a}_2,{b}_2\right)\\& \textbf{Equation 2.0a}\ \textrm{ Beta regression to fit proportion}\\ &\quad\quad\quad\quad\quad\quad\textrm{of adipose that is lipid} \end{align*}

\begin{align*} & \quad \quad\quad\quad logit\left({\mu}_{P_{AL}}\right)=\left(\ {\alpha}_{P_{AL_{\left[ Sex\right]}}}+{\beta}_{P_{AL}} SMI\right)\\& \textbf{Equation 2.0b}\ \textrm{ Logit link to linear regression relating}\\& \textrm{ proportion of adipose that is lipid to body condition} \end{align*}

**Equation 2.0c** First shape parameter for beta regression. \begin{align*} {a}_2={\mu}_{P_{AL}}\ast{\varphi}_2 \end{align*}

**Equation 2.0d** Second shape parameter for beta regression. \begin{align*} {b}_2=\left(1-{\mu}_{P_{AL}}\right)\ast{\varphi}_2 \end{align*}

To determine the proportion of adipose that is protein (*P_AP_*), we quantified the relationship between the proportion of lipid (*P_AL_*) and protein in adipose tissue (Eqn. 3.0a-d, Box 1, see Supplementary Materials 1.5) using the chemical analysis of subcutaneous adipose tissue samples (Supplementary Fig. S3) from the dissection data (Cattet, 1988).

\begin{align*} &\quad\quad\quad\quad\quad\quad\quad{P}_{AP}\sim beta\left({a}_3,{b}_3\right) \\& \textbf{Equation 3.0a}\ \textrm{ Beta regression fitting proportion of} \\ &\text{adipose that is protein given the proportion lipid.}\end{align*}

\begin{align*}& \quad\quad\quad\quad \quad logit\left({\mu}_{P_{AP}}\right)=\left(\ {\alpha}_{P_{AP}}+{\beta}_{P_{AP}}{P}_{AL}\right) \\& \textbf{Equation 3.0b}\ \textrm{ Logit link to linear regression relating} \\ &\textrm{proportion of adipose that is protein to the}\\& \textrm{proportion that is lipid}\end{align*}

**Equation 3.0c** First shape parameter for beta regression. \begin{align*} {a}_3={\mu}_{P_{AP}}\ast{\varphi}_3 \end{align*}

**Equation 3.0d** Second shape parameter for beta regression. \begin{align*} {b}_3=\left(1-{\mu}_{P_{AP}}\right)\ast{\varphi}_3 \end{align*}

Once fit, the equation series 2.0 and 3.0 provide a model to estimate the proportion of adipose that is lipid and protein. When combined with equation series 1.0–1.4 that provide a model to estimate total storage adipose mass and inserting values for lipid and protein energy density (Elia and Cummings, 2007), an estimate for the total energy available in storage adipose can be calculated. We used both weakly informative and unrestricted prior distributions within the Bayesian frameworks to fit all models and compared resulting parameter estimates to assess the influence of priors on the posteriors (Table 1; see Supplementary Materials 4.0 for details).

**Table 1:** Prior distributions assigned to each estimated parameter across all three fitting processes (Box 1). Note that STAN allows improper priors (i.e. Uniform distribution with infinities), and we confirmed that resultant posteriors were indeed proper by comparing to those estimated by the proper prior (i.e. normal distribution around 0)

Parameter	Description	Distribution (unrestricted)	Distribution (weakly informative)
Equation 1.0a: Estimating total muscle mass (U)			
${\alpha}_{lU_0}$	Intercept	Uniform(-Inf, Inf)	Normal(0, 2)
${\beta}_{lU_1}$	Slope from logged length	Uniform(-Inf, Inf)	Normal(0, 2)
${\beta}_{lU_2}$	Slope from logged adipose	Uniform(-Inf, Inf)	Normal(0, 2)
${sd}_{lU}$	Standard deviation around logged total muscle mass	Uniform(0, Inf)	Uniform(0, 1)
Equation 1.1: Estimating mass of structure non-muscle (K_nU_)			
${\alpha}_{lK{ nU}_0}$	Intercept	Uniform(-Inf, Inf)	Normal(0, 2)
${\beta}_{lK{ nU}_1}$	Slope contribution from logged straight-line body length	Uniform(-Inf, Inf)	Normal(0, 2)
${sd}_{lKnU}$	Standard deviation around logged total structure non-muscle mass	Uniform(0, Inf)	Uniform(0, 1)
Equation 1.4: Estimating proportion of storage that is muscle (${\mathrm{P}}_{{\mathrm{O}}_{\mathrm{U}}}$) from body condition			
${\alpha}_{P_{Ou}}$	Intercept	Uniform(-Inf, Inf)	Normal(0, 2)
${\beta}_{P_{Ou}}$	Slope contribution from body condition (SMI)	Uniform(-Inf, Inf)	Normal(0, 2)
${\varphi}_1$	Variance around the proportion of storage that is muscle	Uniform(0, Inf)	Normal(0, 2)
Equation 2.0: Estimating proportion of adipose that is lipid (P_AL_)			
${\alpha}_{P_{AL_{\left[M\right]}}}$	Male specific intercept	Uniform(-Inf, Inf)	Normal(0, 2)
${\alpha}_{P_{AL_{\left[F\right]}}}$	Female specific intercept	Uniform(-Inf, Inf)	Normal(0, 2)
${\beta}_{P_{AL}}$	Slope contribution from body condition (SMI)	Uniform(-Inf, Inf)	Normal(0, 2)
${\varphi}_2$	Variance around the proportion of adipose that is lipid	Uniform(0, Inf)	Uniform(0, 100)
Equation 3.0: Estimating proportion of adipose that is protein (P_AP_)			
${\alpha}_{P_{AP}}$	Intercept	Uniform(-Inf, Inf)	Normal(0, 2)
${\beta}_{P_{AP}}$	Slope contribution from the proportion of adipose that is lipid	Uniform(-Inf, Inf)	Normal(0, 2)
${\varphi}_3$	Variance around the proportion of adipose that is protein	Uniform(0, Inf)	Uniform(0, 100)

Available data on muscle lipid content were limited and showed a weak relationship with protein (Supplementary Fig. S4), so we established the average and upper/lower bounds on the proportion of storage muscle that is protein (*P_UP_*) and lipid (*P_UL_*) from chemical analyses of polar bear biceps femoris muscle (Cattet, 1988; Whiteman *et al.*, 2017) (see Supplementary Materials 1.6). We then explored how using the upper or lower bounds for protein and lipid in storage muscle affected estimates of storage muscle energy.

### Determining the amount of energy in storage mass

Once fit, the model can be used to estimate the mass of structural muscle (*K_U_*; Eqn. 1.0b) and structure non-muscle (*K_nU_*; Eqn. 1.1) from straight-line body length alone. The sum of these provides an estimate for the structural mass (*K*) of an individual polar bear (Eqn. 4.0). Storage mass (*O*) can then be estimated as the difference between total body mass (*M*) and structural mass (*K*) (Eqn. 4.1). The proportion of storage that is muscle (${P}_{O_U}$) can be estimated from non-destructive measurements of body condition (*SMI*; Eqn. 1.4a-d), which, when combined with the equation for the mass of total storage (Eqn. 4.1), enables the prediction of storage muscle (*O_U_*) and storage adipose (*A*) masses (Eqn. 4.2a-b).

**Equation 4.0** Estimate mass of structure. \begin{align*} K={K}_U+{K}_{nU} \end{align*}

**Equation 4.1** Estimate mass of storage. \begin{align*} O=M-K \end{align*}

**Equation 4.2a** Estimate mass of storage muscle. \begin{align*} {O}_U=O\ast{P}_{O_U} \end{align*}

**Equation 4.2b** Estimate mass of storage adipose. \begin{align*} A=O\ast \left(1-{P}_{O_U}\right) \end{align*}

Given the scaled mass index, which only requires total body mass and straight-line body length (Eqn. S3), the masses of lipid and protein in storage adipose can then be predicted by multiplying the mass of storage adipose (*A*) by the model-predicted proportion that is lipid (*P_AL_*; Eqn. 2.0) and protein (*P_AP_*; Eqn. 3.0). Similarly, the masses of lipid and protein in storage muscle (*O_U_*) can be predicted by multiplying the mass of storage muscle by the estimated proportion that is lipid (*P_UL_*; [minimum = 0.0030, average = 0.0218, maximum = 0.0680]) and protein (*P_UP_*; [minimum = 0.2050, average = 0.3738, maximum = 0.6200]). The total mass of lipids (*O_L_*) and protein (*O_P_*) in storage is then estimated as the sum of lipids (Eqn. 5.0) and proteins (Eqn. 5.1) within each storage compartment (i.e. adipose, storage muscle). Finally, total storage energy (*E_O_*) can be estimated by multiplying the storage masses of lipids (*O_L_*) and proteins (*O_P_*) by their respective metabolizable energy content (lipid: ${\varepsilon}_L=39.3\ \frac{MJ}{KG},$and protein:${\varepsilon}_P = 18.4\ \frac{MJ}{KG}$) (Elia and Cummings, 2007) and summing (Eqn. 5.2). Please see Supplementary Materials Section 7 for a complete walkthrough of the multi-storage body composition model calculations for an example bear. To explore how energy estimates were influenced by accounting for storage composition, we calculated total storage energy for hypothetical adult polar bears across a range of straight-line body lengths and total body masses using both the multi-storage model (using average values for the proportions of storage muscle that are lipid and protein, respectively) and the single-storage polar bear body composition model (where for the latter, we assumed model parameters as in Molnár *et al.* (2009), except for the protein energy density, which we updated from ${\varepsilon}_P=18.0\ \frac{MJ}{KG}$ (Molnár *et al.*, 2009; their Fig. 2) to ${\varepsilon}_P=18.4\ \frac{MJ}{KG}$ (Elia and Cummings, 2007) to match the value used here).

**Equation 5.0** Estimate mass of storage lipids. \begin{align*} {O}_L={P}_{AL}A+{P}_{UL}{O}_U \end{align*}

**Equation 5.1** Estimate mass of storage proteins. \begin{align*} {O}_P={P}_{AP}A+{P}_{UP}{O}_U \end{align*}

\begin{align*} &\quad\quad\quad\quad\quad\quad\quad{E}_O={\varepsilon}_L{O}_L+{\varepsilon}_P{O}_P \\& \textbf{Equation 5.2}\ \textrm{ Convert lipid and protein masses to}\\& \text{energy and sum to calculate total storage energy.} \end{align*}

**Figure 2:**
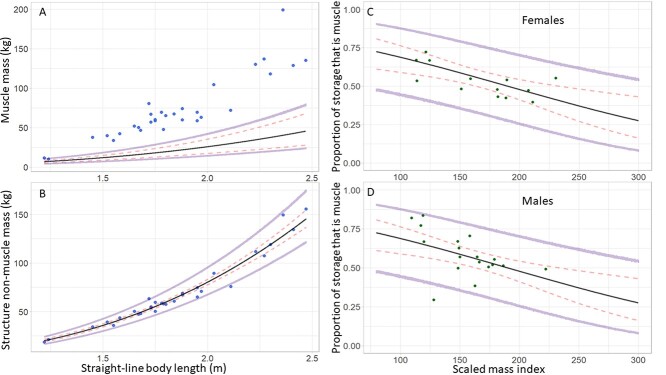
Predicted values for (A) structural muscle (*K_U_*), (B) structure non-muscle (*K_nU_*) and the proportion of storage that is muscle (${P}_{O_U}$) in females (C) and males (D) from the fitted equations of the multi-storage model, together with measurement data from dissected bears. Black lines show the model predictions and colored circles show data for dissected bears. The 95% credible intervals (red dashed lines) show the prediction error associated with parameter estimate uncertainties, and the error associated with the data model variance is shown by the 95% prediction intervals (purple lines). Note that in (A) the black line shows the model-predicted mass of structural muscle, while the blue circles show the observed mass of total muscle (i.e. both structural and storage muscle). In (C) and (D) the green circles show the proportion of storage that is muscle for individual dissected bears, calculated using their observed mass of total muscle and model predicted mass of structural muscle.

### Model testing

Given the small number of dissected bears (*n* = 31), we did not withhold any datapoints during model parameterization to allow model testing against an independent dataset. Instead, we tested the models using the separate isotopic dilution dataset that measured the change in whole body lipids of polar bears (Arnould and Ramsay, 1994; Atkinson *et al.*, 1996; Pagano *et al.*, 2018). The isotopic data were collected from polar bears in the Western Hudson Bay subpopulation (*n* = 32) during 1989–1994 and the Southern Beaufort Sea subpopulation (*n* = 4) during 2014–2016. Western Hudson Bay data were obtained from published studies (Atkinson and Ramsay, 1995; Atkinson *et al.*, 1996), with measurements for a further 16 females obtained directly from the author (S. Atkinson, unpublished data). Data were comprised of adult females with dependent yearlings (*n* = 12) and adult females with dependent cubs-of-the-year (*n* = 13); adult males (*n* = 5; Atkinson *et al.*, 1996); and pregnant adult females that were captured before denning and after parturition (*n* = 2; Atkinson and Ramsay, 1995). Samples were collected in Western Hudson Bay primarily from July—October (with the exception of post parturition captures, which occurred in March), when bears were on land and primarily fasting (Arnould and Ramsay, 1994; Atkinson and Ramsay, 1995). Samples were collected from polar bears on the sea ice in the Southern Beaufort Sea in April, when seal prey was accessible and most bears were feeding (Pagano *et al.*, 2017, 2018). Total body lipid mass was estimated for each individual using isotopic dilution (Farley and Robbins, 1994; Pagano *et al.*, 2017). Sex, age, body mass and straight-line body length were also recorded at each capture. Total body mass was measured using an electronic load cell suspended from an aluminum tripod. All polar bears were captured twice (capture intervals ranged from 8–88 days but were 194 and 200 days for the pregnant females that were measured before and after denning), providing repeated measures of body lipids. All bears lost total body mass between measurements except for two adult females in the Southern Beaufort Sea that gained mass.

Since fully grown bears have stopped depositing structural lipids (e.g. footpads, eye socket) and do not break down structural lipids (unless experiencing severe starvation), the change in lipid mass between two isotopic measurements in adult bears can be attributed to a change in storage lipids. Thus, the difference in lipid mass is comparable to predictions from our model, which predicts only storage lipids. We did not find a significant difference in straight-line body length across the two time points (two-tailed *t*-Test: paired two sample for means *P* = 0.97, df = 35), so we attributed any difference in straight-line body length of the same individual to measurement error, rather than growth, and used the mean straight-line body length for each bear at each timepoint.

We estimated the storage lipid mass for each bear in the isotopic dilution dataset given their straight-line body length and total body mass at each timepoint using the multi-storage body composition model (see **Determining the Amount of Energy in Storage Mass**) and calculated the difference. We first estimated the change in storage lipid mass using the average proportion of lipid observed in muscle, and then also using the minimum and maximum [minimum = 0.0030, average = 0.0218, maximum = 0.0680] to establish uncertainty bounds.

To assess the importance of accounting for storage composition, we also calculated the change in lipids using the single-storage model developed by Molnár *et al.* (2009; their Table 3). We compared the model-derived estimates of change in lipid mass to those measured by isotopic dilution. We used root mean squared error (RMSE) values to compare predictions from each body composition model to the isotopic dilution estimates. We note that the values for the proportion of lipid in storage in the single-storage model (Molnár *et al.*, 2009) were derived from the WH bears in our testing isotopic dilution dataset (i.e. we would expect predictions from the single-storage model to closely match those from the isotopic dilution data for WH bears).

## Results

### Model diagnostics

Diagnostics from all model fits converged well ($\hat{\boldsymbol{R}}$ < 1.0), effective sample sizes were above 100, no iterations ended in divergence, and Bayesian Fraction of Missing Information indicated successful adaptation and efficient exploration of the posterior distribution on all chains for all models (Supplementary Table S5, Supplementary Fig. S6). Parameter estimates were not impacted by different priors (Table 1, Supplementary Table S6-S8). The combined fitting process produced more divergent iterations and a smaller effective sample size - indications of insufficient data or a poorer model fit - when the equation to estimate the proportion of storage that is muscle (Eqn. 1.4b) was allowed to vary by sex (Supplementary Table S5, see Supplementary Materials 1.3.1 and 4.0). Since the model with sex-dependent parameters indicated poor fit, we focus on the results from the model without sex-based differences henceforth. The pairs correlations from the combined fitting process (Eqn. 1.0a-1.4) show a strong correlation between parameters within the same regression but weak correlation between parameters across regressions, indicating that the parameter space was well explored (Supplementary Fig. S6). The estimated parameter values from all fitting processes are reported in the Supplementary Materials (Supplementary Table S6-S8).

**Figure 3:**
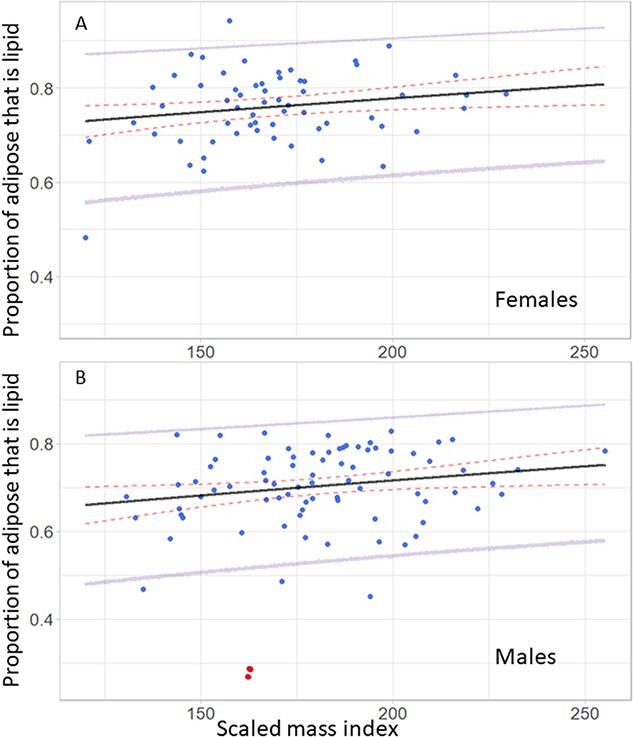
Model-predicted proportion of adipose that is lipid (*P_AL_*; black line) across a range of body conditions as determined by the scaled mass index using the fitted equation 2.0 (Box 1 bottom left), and the observed proportion of adipose that is lipid for each adipose biopsy sample (blue circles) for females (A) and males (B). The 95% credible intervals (red dashed lines) show the prediction error associated with parameter estimate uncertainties, and the error associated with the data model variance is shown by the 95% prediction intervals (purple lines). Two male cubs-of-the-year that were excluded from our analyses are highlighted in red (A).

**Figure 4:**
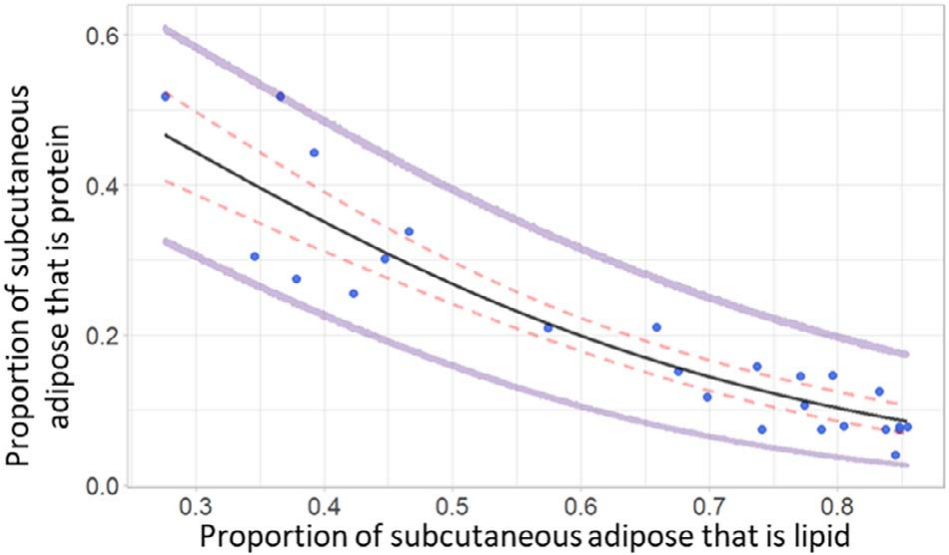
Model-predicted proportion of adipose that is protein (*P_AP_*; black line) given a range of adipose lipid proportions using the fitted equation 3.0 (Box 1 bottom right), and the observed proportion of adipose that is protein for each molecular composition analysis on dissected subcutaneous adipose tissue (blue circles). The 95% credible intervals (red dashed lines) show the prediction error associated with parameter estimate uncertainties; the error associated with the data model variance is shown by the 95% prediction intervals (purple lines).

### Assessing the fit of the multi-storage model and posterior predictions for dissection data

The fitted multi-storage model produced realistic posterior predictions of body composition for the dissection data. The predictions for the mass of structural muscle (fitted Eqn. 1.0b) all fell below the observed mass of total muscle for each of the bears in the dissection data (Fig. 2A). The upper bound of the prediction intervals (accounting for the data model uncertainties), all fell below the observed mass of total muscle (Fig. 2A), except for a single data point (a cub with a relatively small straight-line body length: Supplementary Table S1). All except one of the observed masses of structure non-muscle were within the bounds of uncertainty given by the prediction intervals (Fig. 2B) for estimates from the fitted Eqn. 1.1. In both the models of structural muscle (Eqn. 1.0b) and structure non-muscle (Eqn. 1.1), the estimated coefficient for straight-line body length was close to, but less than three (Supplementary Table S6, Supplementary Fig. S7), the expected scaling exponent for length to structure based on general allometric scaling relationships (Molnár *et al.*, 2009; Kooijman, 2010). The estimated total structural mass across all possible straight-line body lengths for cubs-of-the-year, yearlings, subadults and adults, was less than the total body mass observed in polar bears from Western and Southern Hudson Bay (Supplementary Fig. S10).

The proportion of storage that is muscle (Eqn. 1.4a) declined with increasing body condition (i.e. a change in storage composition that indicates an increase in storage energy). This was the case both when the proportion of storage that is muscle was estimated as the difference in observed total muscle and predicted structural muscle (Eqn. 1.3; Fig. 2C, D green circles) and when predicted by body condition (Eqn. 1.4; Fig. 2C, D black line). Only one datapoint, a male cub of the year (Supplementary TableS1) fell outside the prediction interval for the estimated proportion of storage that is muscle (Eqn. 1.4; Fig. 2D).

The predicted proportion of adipose that is lipid (Eqn. 2.0a) showed a weakly positive relationship with body condition (Fig. 3). The intercept for females was higher than for males, leading to higher predictions for proportion of adipose that is lipid for females given the same body condition values as males. Although most observed values fell within the prediction intervals, the upper and lower intervals differ by nearly 35% (Fig. 3). The proportion of adipose that was protein showed an exponential-decay-like relationship with the proportion of adipose that is lipid (Eqn. 3; Fig. 4), and all observed values were within the prediction intervals from the fitted equation.

### Effects of using maximum or minimum reported proportions of lipid and protein in storage muscle

The amount of energy in storage muscle increased by 262% when the maximum proportions of lipid and protein were taken compared to the minimums (Supplementary Table S3). When the proportion of lipid was held constant at its minimum, increasing the minimum protein estimate to the maximum increased energy content by 196%, and increased by 118% when the proportion of lipid was held constant at its maximum (Supplementary Table S3). When the proportion of protein was held constant at its minimum, increasing the minimum lipid estimate to the maximum increased energy content by by 66%, and increased by 22% when the proportion of protein was held constant at its maximum (Supplementary Table S3). Despite the lower energetic value of protein compared to lipid, the larger variation in observed muscle protein content resulted in larger variations in total energy of muscle tissue.

### Effects of accounting for storage composition on storage energy estimates

**Figure. 5:**
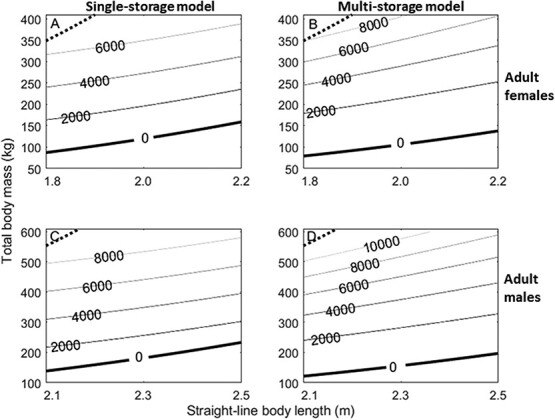
Total storage energy (MJ) predicted by the single-storage body composition model (A, C) and the multi-storage compartment body composition model (B, D) developed here. Storage energy is calculated for hypothetical polar bears across a range of feasible straight-line body lengths and total body masses for adult females (A, B) and adult males (C, D) using parameter values calculated where the proportion of storage that is muscle was calculated without any sex-related differences (Eqn. 1.4a; Box 1) and weakly informative priors. Minimum (solid thick black) and maximum (dotted black) combinations of mass and length are shown. Minimums are calculated as the structural mass estimates from each respective model, while approximate maxima are calculated as four times the structural mass—as defined by the single-storage model—in the same way as Molnár *et al.* (2009).

**Figure 6:**
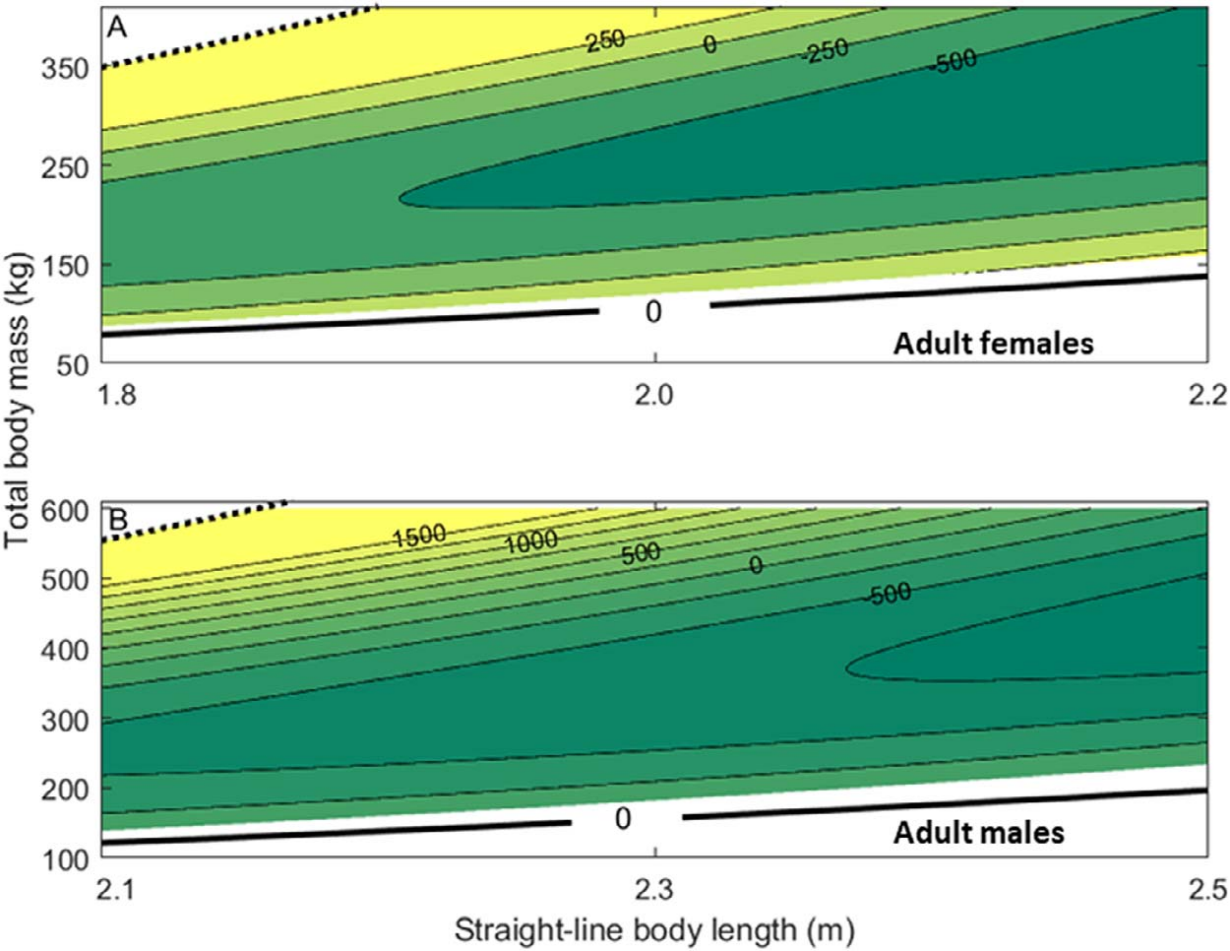
The difference in total storage energy (MJ) predicted by the single-storage compartment body composition model and the multi-storage compartment body composition model. The difference in storage energy estimates is shown for hypothetical polar bears across a range of feasible straight-line body lengths and total body masses for adult females (A) and adult males (B). Difference in storage energy predictions is calculated as the multi-storage estimate minus the single-storage estimate (contour lines), so that negative values indicate that the single-storage model predicted higher storage energy. Darker colors indicate larger differences where the single-storage model predicts higher energy. Minimum (solid thick black) and maximum (dotted black) combinations of mass and length are shown. Minima are calculated according to the structural mass from the multi-storage model, with the white area indicating mass-length combinations that are possible with the multi-storage model but are deemed impossible with the single-storage model due to total body mass falling below the predicted structural mass. Maximums are calculated as four times the structural mass—as defined by the single-storage model—in the same way as Molnár *et al.* (2009).

The estimates for total storage energy from the single-storage and multi-storage body composition models were similar across the range of hypothetical length and mass values (Fig. 5). However, when comparing storage energy estimates for a given straight-line body length, energy estimates from the multi-storage model increased more rapidly than those from the single-storage model given the same unit increase in mass (i.e. with increasing mass, the spacing between contour lines remains roughly the same in the single-storage model but decreases in the multi-storage model) (Fig. 5). In both males and females, predicted energy stores were higher from the single-storage model across all lengths at lower masses but this pattern flipped as mass increased (e.g. for a female of 1.8 m length, the single-storage model predicted 2000 MJ in storage at a mass of ~ 163 kg, while the multi-storage predicted 2000 MJ at a mass of ~ 179 kg; whereas 6000 MJ was predicted at body masses of 317 kg and 299 kg by the single-storage and multi-storage models, respectively; Figs 5, 6). The multi-storage model also predicted higher total energy near the minimum body mass values, a trend that was more prominent in females (Fig. 6).

For both males and females, the structural mass estimated by the multi-storage model was lower than the estimate from the single-storage model (Figs 5, 6). The multi-storage model also predicted that the shape-density parameter, which relates length to structural mass, decreased with increasing length (Supplementary Fig. S11).

### Model testing

**Figure 7:**
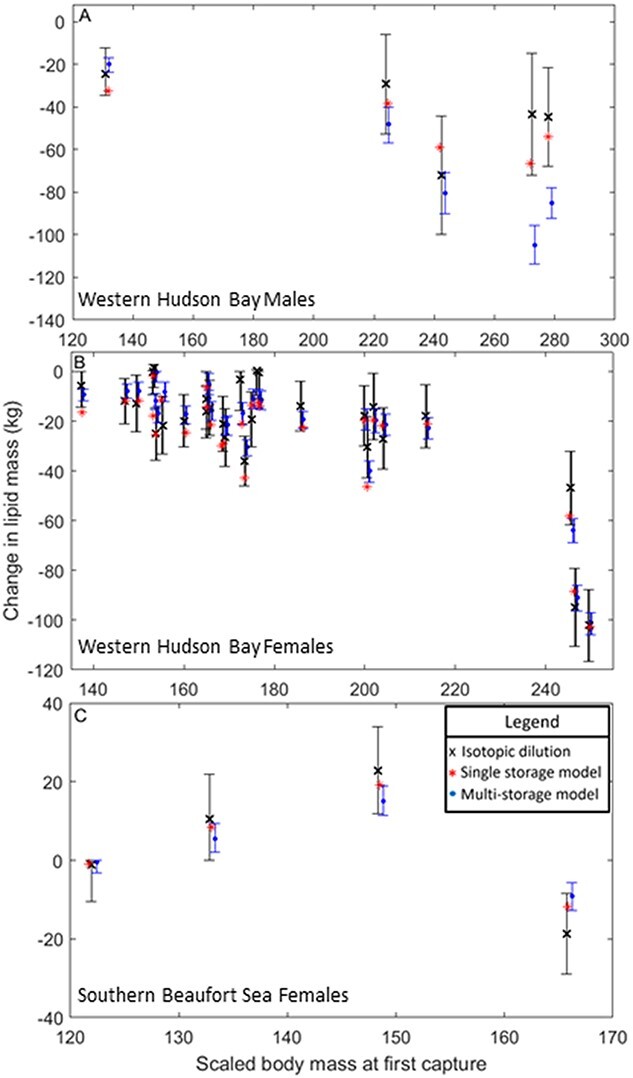
Predictions for the change in lipid mass between repeated measurements for adult polar bears according to isotopic dilution (black cross), the single-storage compartment body composition model (red star), and the multi-storage compartment body composition model (blue circle) developed here. Panel (A) shows predictions for adult males in the Western Hudson Bay subpopulation; panel (B) shows predictions for adult females in the Western Hudson Bay subpopulation; and panel (C) shows predictions for adult females in the Southern Beaufort Sea subpopulation. The error bars associated with isotopic dilution measurements show the 2.7% error associated with isotopic dilution measurements in bears (Atkinson and Ramsay, 1995). The estimates for the multi-storage model are calculated using parameter values from the base model structures (Box 1) and weakly informative priors. The error bars on the multi-storage model values were calculated using the maximum and minimum values for observed lipid content in muscle tissue. Isotopic dilution measures are located along the exact scaled body mass measures while the estimates from both body composition models are offset to increase readability.

The changes in lipid mass predicted by the multi-storage model fell within the bounds of uncertainty around those predicted by isotopic dilution for all but two WH males (Fig. 7A) and two WH females (Fig. 7B; with scaled mass index at first capture of 155.25 and 153.17). If we consider the error bounds around the multi-storage model estimates, which come from using the minimum and maximum values for protein in storage muscle tissue, the bounds of uncertainty overlapped with all isotopic dilution estimates except the two WH males (Fig. 7). The estimates for the change in lipid mass from the single-storage model (Molnár *et al.*, 2009) fell within the bounds of uncertainty for all isotopic dilution measurements, except for two WH females (Fig. 7).

According to RMSE, the average differences between the model estimated change in lipid mass and that measured by isotopic dilution were 9.2 kg for the single-storage model and 14.5 kg for the multi-storage model, 13.7 kg and 34.1 kg, respectively, when only considering WH males, 4.0 kg and 6.7 kg, respectively, when only considering SB females, and 8.7 kg and 7.7 kg when only considering WH females. To explore where the multi-storage model predictions diverged from the isotopic dilution estimates, we also compared model predictions for a subset of data that excluded the two male polar bears in the best body condition, where the largest discrepancies in predictions were observed (Fig. 7A). Using this subset, predictions for males from the multi-storage model were closer to the isotopic dilution estimates (RMSE: 12.2 kg) and there was a small improvement in the single-storage model RMSE value (RMSE: 10.2 kg). The single-storage model improved slightly (RMSE: 8.5 kg) and the multi-storage model improved considerably (RMSE: 8.1 kg) when considering all bears from the subset. When using the lower bounds on the regression parameters used to estimate the proportion of adipose that is lipid, the multi-storage model predictions for males were more like the isotopic dilution estimates (RMSE = 21.0 kg), resulting in closer predictions for all bears combined (RMSE = 11.1 kg), but divergent predictions for females (RMSE: WH = 8.6 kg, SB = 7.9 kg) (Supplementary Fig. S12). When comparing predictions between the single- and multi-storage models, the RMSE value was 10.4 kg across all bears, 25.1 kg for males, 2.9 kg for SB females, and 5.1 kg for WH females. Using the lower bounds decreased the predicted proportion of adipose that is lipid in the multi-storage model, effectively decreasing the estimated change in lipid mass associated with the observed change in total body mass (Figs 7, Supplementary Fig. S12).

## Discussion

Connecting body condition to underlying body composition, and thus, energy stores, is a critical component of understanding polar bear energetics and predicting impacts from changes in prey availability, such as those due to sea ice loss (Molnár *et al.*, 2010, 2020). Our study used polar bear dissection data to create a body composition model with multiple storage compartments, linking non-destructive measurements of individual straight-line body length and total body mass to underlying lipid and protein stores. We tested the model by predicting changes in lipid mass of wild polar bears using repeated measures of length and mass in recaptured individuals. Overall, the multi-storage model produced similar predictions of lipid mass changes compared to those measured directly using isotopic dilution. Our model builds on and expands an earlier polar bear body composition model (with a single storage compartment) (Molnár *et al.*, 2009), allowing metabolizable lipid and protein energy stores in storage muscle and storage adipose tissue to be quantified, and providing greater flexibility for modelling bioenergetic processes.

Structural masses predicted by the multi-storage model were lower than previous predictions by the single-storage model (Supplementary Figs S10, S11) (Molnár *et al.*, 2009), which contributed to the resultant differences in predicted energy stores between the two models (Figs 5, 6). When testing each model’s ability to predict lipid changes, the multi-storage model performed slightly better when considering only WH females (Fig. 7) (even when considering only those which the single-storage model was trained on), while the single-storage model performed better when applied to SB females. Nonetheless, the differences in lipid predictions between multi- and single-storage models were relatively small for both subpopulations of adult females (~2 kg of lipid or 78.6 MJ), indicating both models were able to give reasonable estimates of lipid change. The difference in predictions for the WH males (a difference of ~ 25 kg of lipid or 982.5 MJ between models) drove the overall discrepancy between the two body composition model estimates. Given general estimates for the daily mass loss in fasting polar bears (Pilfold *et al.*, 2016), the difference in estimated lipid mass for females would lead to a difference of 1–3 days survival, but survival predictions for males during the fasting season could differ by more than 20 days, with the single-storage model predicting faster starvation times. The discrepancy between model estimates for males was halved (~12 kg of lipid or 471.6 MJ) when using the lower bounds of parameter estimates to calculate the proportion of adipose that is lipid (Eqn. 1.4) in the multi-storage model, but this caused increased divergence in estimates for females between models. Furthermore, when the two males in extremely good body condition were excluded, all multi-storage model predictions closely matched those from isotopic dilution. Thus, it appears that the multi-storage model overestimated the increase in either the proportion of adipose that is lipid or the proportion of storage that is adipose (i.e. a higher estimate for total lipids stemming from a higher proportion of adipose), with increasing body condition in males. Given our limited data, explorations into sex-related differences between muscle retention with increasing adiposity were not resolved (Supplementary Table S5) but could play a role in driving higher lipid content in females, which may require substantially more energy for reproduction (Gittleman and Thompson, 1988) due to the demands of parturition and milk production. The ability of females to increase adipocyte volume (Ramsay *et al.*, 1992) is a likely mechanism to allow higher lipid content in females than males at better body condition values, but body condition indices that rely solely on total body mass and straight-line body length may not detect the intricacy of adipocyte dynamics.

We also note that the duration between captures and, thus, isotopic dilution measures, was highest in the two males (88 and 86 days) with the biggest discrepancy between multi-storage and isotopic lipid mass estimates, indicating that the model might perform best over shorter timescales of energy change. Despite the comparatively poorer performance of the multi-storage model for males, the model produced overall similar estimates of lipid change compared to isotopic dilution, suggesting that that the multi-storage model could nevertheless be useful for estimating fine scale body composition changes over smaller temporal scales, or when composition of storage may be liable to change (e.g. during food deprivation, or refeeding).

When considering applications for different body composition models, it is important to note that measurements obtained via isotopic dilution describe whole-body lipids. Isotopic dilution is a valuable means of assessing changes in body composition through time as inclusion of both structural and storage lipids may be of little concern when assessing relative body condition of bears or comparing temporal changes in lipid mass, but such estimates could potentially be less accurate if used as a proxy for the storage energy available for mobilization. As such, the multi-storage model developed here offers a potential means to estimate an individual’s storage lipids (and storage protein), as an alternative to existing body composition models that provide total lipid estimates, but which likely include some structural lipids (i.e. both isotopic dilution and the single-storage model assume all lipids are metabolically available). Moreover, obtaining accurate isotopic dilution measurements can be more complicated if individuals are feeding and have material in their digestive tracts, which can be difficult to account for in the field (Pagano *et al.*, 2017) and which may also be a confounding issue when applying storage composition model estimates without knowing, and accounting for, stomach content. Although relationships between body water and body lipids (that form the basis to isotopic dilution) have been estimated empirically in terrestrial bears (Farley and Robbins, 1994), polar bears may have diverged from the body composition patterns observed in terrestrial ursids (Cattet *et al.*, 2001, 2002). This may further explain small differences between the estimates of lipid change produced by the multi-storage model and isotopic dilution estimates.

A particular advantage associated with both the single and multi-storage models is that the only data required to estimate individual body composition are the more broadly available measurements of total body mass and length. Although training is required to sample polar bears for the collection of mass and straight-line body length accurately, these simple morphometrics are often collected as part of population monitoring programs and can be relatively easily obtained in the field (often in remote and/or challenging conditions) compared to the additional time, skills, and costs typically required for body composition measurements via isotopic dilution. We note that field measurements themselves (particularly length) vary in precision (Rode *et al.*, 2020), adding an additional source of uncertainty. Furthermore, polar bears are often kept under anesthesia longer to collect isotopic dilution data, risking more adverse impacts upon the individual (Arnemo *et al.*, 2006). Despite their divergent assumptions, data requirements, and model structures, the reasonably similar predictions made by the three models examined here highlight that model choice should be informed by the specific research goals and will likely depend on the data and resources available (Pagano *et al.*, 2017).

Across all body composition models, developing a better understanding of the body composition of polar bears and potential variation among individuals is crucial to improving our ability to accurately quantify energy stores. For accurate prediction, body composition models rely on sufficient experimental/empirical data for parameterization and testing. Isotopic dilution and chemical composition data are invaluable for testing and improving the accuracy of storage energy models. In turn, the quantitative models can point to new avenues for data collection, or areas where our understanding is still lacking. For example, a large body of polar bear literature relies on body composition measurements and patterns of energy use observed in a small number of polar bears sampled via isotopic dilution techniques in the 1980s and 1990s (Arnould and Ramsay, 1994; Atkinson and Ramsay, 1995; Atkinson *et al.*, 1996). However, in these studies, cross-sectional data for females only includes females with cubs or pregnant females entering the den and is thus likely skewed towards females that were in relatively good body condition, i.e. insufficient condition to reproduce (Derocher *et al.*, 1993; Molnár *et al.*, 2011). Patterns of energy use or body composition may differ for individuals in poor body condition. For example, the relative use of lipid and protein for energy in male polar bears appears to depend on an individual’s relative body condition at the beginning of the fast (Atkinson *et al.*, 1996), which corresponds with findings in other mammals under controlled studies and species that undergo seasonal fasting periods (Cherel *et al.*, 1992, 1993). Reliable predictions for survival and reproduction under novel environmental conditions require more data to improve our understanding of energetic processes, and the factors driving the molecular composition of polar bear storage mass. In our study, the extreme difference in storage muscle energy when estimated using maximum versus minimum observed proportions of protein and lipid in muscle tissue (262% change in energy, which translates to over 1000 MJ in some individuals, Supplementary Table S3) exemplifies the impact of uncertainties surrounding molecular composition. The collection and analysis of non-destructive muscle and adipose tissue biopsies for protein and lipid content, in tandem with total body mass and length measures, can provide insight on the mechanisms driving the energy content of these storage tissues beyond body condition. Promoting the opportunistic collection of more dissection data through collaborations with hunters and managers (e.g. when euthanizing problem bears), and more extensive reporting of mortalities could aid in refining body composition models. The use of captive polar bears could also increase data collection and model testing. The development of novel techniques and sampling strategies (e.g. crate training with captive bears (Penk, 2021)) can allow procedures such as isotopic dilution to be performed under controlled settings, whereby changes in body composition and energy storage can be related to known amounts of assimilate (i.e. meals) and energy expenditures.

The multi-storage model developed here can advance bioenergetic modelling approaches (e.g. dynamic energy budget (DEB) modelling) that require estimation of body composition and energy stores. DEB modelling relies on accurate representation of energy assimilation and use at the individual level, where fluctuations in energy stores can indicate the energetic cost of biological processes such as basal metabolism, reproduction, and locomotion (Molnár *et al.*, 2009; Kooijman, 2010). The single-storage model was originally developed for integration with DEB models (Molnár *et al.*, 2009) to provide estimates of structural mass and storage mass and has been applied to quantify energy dynamics of fasting bears (e.g. Molnár *et al.*, 2010, 2011, 2020). However, the differential accumulation/depletion of protein/lipid observed in polar bears of different body conditions (Atkinson *et al.*, 1996; Pagano *et al.*, 2018) suggests that polar bears may not always meet the DEB assumption of single compartment homeostasis (i.e. that the molecular composition within the structural and storage compartments remains in equilibrium regardless of overall mass changes; Kooijman, 2010). The multi-storage model addresses potential variation in polar bear storage composition by further dividing the storage compartment into multiple sub-compartments to establish more fine scale estimates of lipid and protein masses (Eqn. 5.0, 5.1), such that the relative proportions of each can change, which has been previously accomplished for invertebrate species (Kuijper, 2004; Venolia *et al.*, 2020). Furthermore, the delineation of storage into distinct energy sources enables more fine-scale separation of resource assimilation and resource mobilization in bioenergetic modelling (Kooijman, 2010; Sousa *et al.*, 2010).

By estimating fine scale body composition of bears, the multi-storage model can enable the expansion of existing polar bear bioenergetic models beyond fasting seasons to periods when significant feeding is occurring by allowing the amount of lipid and/or protein from different meal types (e.g. seal muscle versus seal blubber) to be explicitly accounted for. The separation of energy sources also enables different proportions of each to be used for different metabolic processes, which could potentially assist in modelling reproductive costs, for example, if the energy required for lipid-rich milk (Derocher *et al.*, 1993; Arnould and Ramsay, 1994) disproportionately depletes more lipid than protein compared to other energetic processes, or if females minimize protein catabolism when denning (Oftedal, 2000). The discrete values estimated for the average proportion of lipid in the single-storage model for different age classes (Molnár *et al.*, 2009) can present a problem for modelling continuous changes in storage composition with age. The multi-storage model circumvents this issue by dynamically calculating the proportion of lipid in storage based on an individual’s body composition across all ages. However, caution should be taken when using the multi-storage model to est energy stores in young bears given the small sample size of dissected dependent cubs (Supplementary Table S1). Similarly, the same principles of continuous composition changes could be applied to structural components of polar bears to account for age-related changes in the molecular composition of structural mass (Clarke, 2008), which is currently unknown but could play a role in structural growth. The decreasing value of the shape-density parameter that relates straight-line body length to total structural mass (Supplementary Fig. S11) suggests that the assumption of isomorphy in the single-storage model, may not be suitable for polar bears. However, the difference is most drastic for straight-line body lengths < 1.0 m (Supplementary Fig. S11), which was outside the range of the dissection dataset. Regardless, care should be taken in assuming isomorphy of structure as this could lead to inaccurate calculations of storage mass. Further resolution could perhaps be attained by looking at shape in a more abundant, closely related species (e.g. black bears (*Ursus americanus*) and brown bears (*Ursus arctos*)) to determine if there is evidence for structural shape change through ontogeny.

The multi-storage body composition model for polar bears can expand our ability to understand and predict population dynamics. The polar bear dissection and chemical extraction data used to develop the multi-storage model are the most accurate and detailed available to date, thus replacing single-storage model assumptions, such as storage molecular composition, with mechanistic underpinnings based on body condition. The multi-storage model can act as a complementary tool to existing body composition models to expand the range of conditions under which estimates of energy stores can be made and further account for the nuanced mechanisms that drive individual energy stores. The separation of storage into muscle and adipose—and the ability to estimate the proportion of protein and lipid within each—not only enables dynamic estimates of storage tissue energy density, but also facilitates the expansion of the polar bear life cycle that can be modelled. Improving our ability to accurately quantify the storage energy of polar bears will contribute to our understanding of the energy dynamics of individuals across their life cycle. Subsequently, our current understanding of how animals respond to and recover from prolonged food deprivation can be tested and built upon, thus enhancing our ability to forecast how population vital rates will respond to novel environmental conditions. The modeling framework presented here is broadly applicable to other species where the composition of energy storage is likely to vary, providing an approach to build more physiologically complex animal bioenergetic models.

## Author Contributions

S.R.P and P.K.M conceived the study. All authors contributed expertise to discussions on model development. S.R.P and P.S performed all analyses involved in the model development. S.R.P performed the model testing. S.R.P, P.S, and L.C.A developed the first draft of the manuscript. All authors contributed to evaluating and editing the manuscript. M.R.L.C, G.W.T, N.J.L and A.M.P provided data used in the study.

## Funding

This work was supported by Polar Knowledge Canada from the Northern Scientific Training Program grant which was awarded to S.R.P. L.C.A was supported by a Mitacs Elevate Postdoctoral Fellowship and Polar Bears International. The work was supported by a Natural Sciences and Engineering Research Council of Canada (NSERC) Discovery Grant (RGPIN-2016-06301), the Canada Foundation for Innovation (CFI) John R. Evans Leaders Fund (35341), and the Ministry of Research, Innovation and Sciences (MRIS) Ontario Research Fund which were awarded to P.K.M. All data contributed by M.R.L.C was supported by the Boreal Institute for Northern Studies (since dissolved), Environment and Climate Change Canada (Canadian Wildlife Service), Natural Sciences and Engineering Council Canada, Environment and Climate Change (NWT), and Polar Continental Shelf Project. Research in the Southern Beaufort Sea was funded by the U.S. Geological Survey Changing Arctic Ecosystems Initiative and the Species and Land Management programs of the U.S. Geological Survey Ecosystems Mission Area.

## Data Availability

No new data were collected for this study. We have published the tissue masses and compositions from dissected polar bears along with the manuscript. Average and ranges from the molecular analyses of different tissue types are given in the Supplementary Materials (Supplementary Table S2) along with figures depicting the protein (plus ash) and lipid content in adipose and muscle tissue (Supplementary Figs S3, S4), and the raw data will be provided to interested parties upon reasonable request. The raw adipose tissue biopsy dataset is available upon request and the percentage lipid content data are graphically displayed in the Supplementary Materials (Supplementary Fig. S5). Most of the Western Hudson Bay bear data from the isotopic dilution dataset are available in the published literature (Arnould and Ramsay, 1994; Atkinson and Ramsay, 1995) while the data on Southern Beaufort Sea bears can be found as a U.S. Geological Survey data release in Pagano *et al.* (2018). The complete isotopic dilution dataset used in model testing, including the unpublished data from S. Atkinson, is available upon request. The total body mass and straight-line body length of Western Hudson Bay polar bears used to develop the scaled mass index is graphically displayed in the Supplementary Materials (Supplementary Fig. S1) and can be made available in an Excel file upon request.

## Supplementary Material

Web_Material_coad043
