# Supplementary material for: A body composition model with multiple storage compartments for polar bears (Ursus maritimus)
Source: Conserv Physiol. 2023 Jun 20;11(1):coad043. doi: 10.1093/conphys/coad043 (PMC10281502; doi:10.1093/conphys/coad043)
Supplement: Web_Material_coad043 [file web_material_coad043.zip › Supplementary_Materials_for_Penk_et_al_Polar_bear_body_composition_Revision_final (3).pdf]

A body composition model with multiple storage compartments for polar bears (*Ursus maritimus*)

Stephanie R. Penk[1,2]; https://orcid.org/0000-0002-8027-4372

Pranav Sadana[1,3]; https://orcid.org/0000-0002-4277-4755

Louise C. Archer[1]; https://orcid.org/0000-0002-1983-3825

Anthony M. Pagano[4]; https://orcid.org/0000-0003-2176-0909

Marc R. L. Cattet[5]; https://orcid.org/0000-0002-2318-1452

Nicholas J. Lunn[6]; https://orcid.org/ 0000-0003-0189-5494

Gregory W. Thiemann[7]; https://orcid.org/ 0000-0002-1888-900X

Péter K. Molnár[1,2]; https://orcid.org/0000-0001-7260-2674

[1] Laboratory of Quantitative Global Change Ecology, Department of Biological Sciences, University of Toronto Scarborough, 1265 Military Trail, Scarborough, ON M1C 1A4, Canada

[2] Department of Ecology and Evolutionary Biology, University of Toronto, 25 Willcocks Street, Toronto, ON, M5S 3B2 Canada

Corresponding author. E-mail: sr.penk@mail.utoronto.ca

[3] Department of Biology, University of Winnipeg, 515 Portage Ave, Winnipeg, MB R3B 2E9, Canada

[4] U.S. Geological Survey, Alaska Science Center, Anchorage, Alaska, 99508 USA

[5] Western College of Veterinary Medicine, University of Saskatchewan, 52 Campus Dr, Saskatoon, SK S7N 5B4, Canada

[6] Wildlife Research Division, Science and Technology Branch, Environment Canada and Climate Change Canada, Edmonton, Canada

[7] Faculty of Environmental and Urban Change, York University, 4700 Keele St., Toronto ON M3J 1P3

Supplementary Materials

1 Model Development Details 3
1.1 Developing the Equations to Estimate the Masses of Structural Muscle and Structure Non-muscle 3
1.2 Developing the Equations to Estimate the Proportion of Storage that is Muscle 4
1.3 Developing the Equation to Estimate Storage Composition given Body Condition..........................5
1.3.1 Model Structure Test 5
1.4 Developing the Equation to Estimate the Proportion of Adipose that is Lipid 6
1.5 Developing the Equation to Estimate the Proportion of Adipose that is Protein 7
1.6 Estimating the Proportion of Storage Muscle that is Lipid and Protein 8
2 Developing the Scaled Mass Index 9
3 Dataset Details..15
3.1 Dissected Bear Data 15
3.2 Adipose Biopsy Data 19
4 Priors and Model Metrics 21
5 Parameter Estimates..26
6 Model Testing 28
7 Implementing the Multi-Storage Model...34
8 References 37

1 Model Development Details

1.1 Developing the Equations to Estimate the Masses of Structural Muscle and Structure Non-muscle

We estimated the masses of structure non-muscle and structural muscle by constructing three log-log linear models. Models were fit simultaneously, thus allowing us to estimate latent variables (i.e., unobservable variables that must be inferred through a model) while using them within the combined fitting process (Box 1 top panel). The first statistical model predicts logged total muscle mass (U) from the natural logs of straight-line body length (L) and adipose tissue mass (A) (Eqn. 1.0a). We expected that structural muscle mass depends on the size of structure (a larger skeletal frame requires more structural musculature/connective tissue), while storage muscle mass depends on adipose tissue mass (because additional muscle must be built to support and move adipose tissue). Thus, we assumed total muscle mass would be related to both L (which is a strong predictor for skeletal mass in polar bears (Cattet *et al.*, 2002)) and A, so included both variables as predictors in the model (Box 1; Eqn. 1.0a). We took the log of all variables for this analysis because all variables were positive and we expected relationships to muscle mass to be potentially non-linear and multiplicative with respect to L and A (Gelman and Hill, 2006). The second statistical model (Box 1, Eqn. 1.0b) estimated the mass of structural muscle (K_U) by using the coefficients from fitted Eqn. 1.0a to predict muscle mass for each datapoint when adipose was set to zero (i.e., setting $A = 0$ in Eqn. 1.0a, meaning that only muscle associated with the structural predictor L was considered). To predict the remaining structural mass (K_{nU}) (i.e., structure non-muscle), and thus enable estimation of the entire structural mass (K; Fig. 1), a third log-linear model estimated the mass of structure non-muscle (K_{nU}) from straight-line body length L (Eqn. 1.1). Both the logged mass of muscle and logged mass of

structure non-muscle were fit from independent normal distributions (Box 1, Eqn. 1.0a, 1.1) to estimate the intercept (α_{lU_0}, α_{lKnU_0}) and slopes (β_{lU_1}, β_{lU_2}, β_{lKnU_1}) for the predictor variables in each regression, as well as the standard deviation around both outcome variables (sd_{lU}, $sd_{lK_{nU}}$). These regression equations are restated below for convenience, and are the same as in the main text. Prior distributions for the Bayesian framework are as described in Table 1.

$\log(U) \sim N(\alpha_{lU_0} + \beta_{lU_1}\log(L) + \beta_{lU_2}\log(A)\,, sd_{lU})$ **Equation 1.0a** Regression to fit total logged muscle

$\log(K_U) = \alpha_{lU_0} + \beta_{lU_1}\log(L) + 0$ **Equation 1.0b** Estimate logged structural muscle

$\log(K_{nU}) \sim N(\alpha_{lKnU_0} + \beta_{lKnU_1}\log(L)\,, sd_{lK_{nU}})$ **Equation 1.1** Regression to fit logged structure non-muscle

1.2 Developing the Equations to Estimate the Proportion of Storage that is Muscle

Next, we determined how the ratio of muscle to adipose in storage mass (Fig. 1) varies with body condition (i.e., we allowed the composition of storage mass to vary depending on body condition). Within the combined fitting process, we estimated the mass of storage muscle O_U (Box 1, Eqn. 1.2) as the difference in the observed total muscle (U) and the estimated structural muscle (K_U, given by Eqn. 1.0b). Thus, O_U was estimated for each parameter combination being sampled at each iteration of the Hamilton Monte Carlo sampling procedure. Using the estimate for storage muscle given by Eqn. 1.2, we then calculated the proportion of storage that was muscle P_{O_U} (Eqn. 1.3), where total storage mass was estimated as the sum of adipose A and estimated storage muscle O_U (Box 1, Fig. 1).

$O_U = U - exp(\alpha_{lU_0} + \beta_{lU_1}\log(L))$ **Equation 1.2** Estimate storage muscle given Eqn. 1.0b

$P_{O_U} = \frac{O_U}{(A+O_U)}$ **Equation 1.3** Estimate proportion of storage that is muscle given Eqn. 1.2

1.3 Developing the Equation to Estimate Storage Composition given Body Condition

The estimated proportion of storage that was muscle (P_{O_U}) derived from the dissection data were used as the dependent variable to simultaneously fit a beta regression model with a logit link equation where scaled mass index (*SMI*) is the predictor variable, thus accounting for the influence of body condition on the ratio of storage muscle to adipose (Box 1, Eqn. 1.4a-b). To test for sex-related differences in how body condition relates to the ratio of muscle and adipose within storage, we evaluated how the data fit three different model structures as follows: 1) parameters were not allowed to vary by sex (Box 1, Eqn. 1.4a-b), a multi-level model (see 'Model Structure Test') that allowed 2) intercept (Eqn. S1a, S2a) and 3) slope to vary by sex (Eqn. S1b, S2b). The beta distribution is parameterized using the shape parameters; thus, we related the mean ($\mu_{P_{Ou}}$) and precision (φ_1) to the shape parameters (a_1, b_1) for input into the beta distribution (Box 1, Eqn. 1.4c-d) (Branscum *et al.*, 2007). Parameter prior distributions for the Bayesian framework are described in Table 1.

$$P_{O_U} \sim beta(a_1, b_1)$$

Equation 1.4a Beta regression to fit proportion of storage that is muscle

$$logit\left(\mu_{P_{Ou}}\right) = \left(\alpha_{P_{Ou}} + \beta_{P_{Ou}} SMI\right)$$

Equation 1.4b Logit link to linear regression relating proportion of storage that is muscle to body condition

$$a_1 = \mu_{P_{Ou}} * \varphi_1$$

Equation 1.4c First shape parameter for beta regression

$$b_1 = (1 - \mu_{P_{Ou}}) * \varphi_1$$

Equation 1.4d Second shape parameter for beta regression

1.3.1 Model Structure Test

The combined model fitting process was first fit assuming no differences across sex (Box 1 top, Eqn. 1.4). To explore potential differences between the sexes, we also implemented a multi-level model framework to allow the proportion of storage that is muscle (Eqn. 1.4) to vary

by sex. To do so, we modified Eqn. 1.4b in two ways: 1) allowing the intercept to vary by sex (Eqn. S1a), and 2) allowing the slope relating *SMI* to the proportion of storage that is muscle to vary by sex (Eqn. S1b).

$$logit(\mu_{P_{Ou}}) = \left(\alpha_{P_{Ou_{[Sex]}}} + \beta_{P_{Ou}} SMI\right) \text{ Equation S1a}$$

$$logit(\mu_{P_{Ou}}) = \left(\alpha_{P_{Ou}} + \beta_{P_{Ou_{[Sex]}}} SMI\right) \text{ Equation S1b}$$

To prevent the addition of extra parameters, as would be the case with completely unpooled data, we used a hierarchical modelling framework such that parameters are drawn from the same normal distribution dictated by hyperparameters (Gelman and Hill, 2006). In both model structures, the hyperparameter mean ($\mu_{\alpha_{Ou}}$or $\mu_{\beta_{Ou}}$) prior is a normal distribution with mean 0 and standard deviation of 1 while the standard deviation ($\sigma_{\alpha_{Ou}}$ or $\sigma_{\beta_{Ou}}$) is drawn from a uniform distribution between 0 and 1 (Eqn. S2a, S2b).

$$\alpha_{P_{Ou_{[Sex]}}} \sim Normal(\mu_{\alpha_{Ou}}, \sigma_{\alpha_{Ou}}) \text{ Equation S2a}$$

$$\beta_{P_{Ou_{[Sex]}}} \sim Normal(\mu_{\beta_{Ou}}, \sigma_{\beta_{Ou}}) \text{ Equation S2b}$$

1.4 Developing the Equation to Estimate the Proportion of Adipose that is Lipid

To determine how much lipid energy was available in storage, we first calculated the amount of lipid in storage adipose. We used the adipose tissue biopsy data from polar bears (Sciullo *et al.*, 2016) to fit the best model to estimate the proportion of lipid in adipose tissue from body condition. We created a multilevel generalized linear model (logit link, beta distribution) (Branscum *et al.*, 2007) that uses the proportion of lipid in polar bear adipose tissue biopsy as a

response variable and each individual's body condition (*SMI*) as the predictor variable (Box 1). We compared the fit from different model structures, which targeted potential sex-related differences (Table S4). The best fit model allows the intercept ($\alpha_{P_{AL}}$) to vary by sex (*Sex*) while slope ($\beta_{P_{AL}}$) remains constant (Box 1, Eqn. 2.0b). Priors for parameters are listed in Table 1.

$$P_{AL} \sim beta(a_2, b_2)$$

Equation 2.0a Beta regression to fit proportion of adipose that is lipid

$$logit(\mu_{P_{AL}}) = \left(\alpha_{P_{AL[Sex]}} + \beta_{P_{AL}} SMI \right)$$

Equation 2.0b Logit link to linear regression relating proportion of adipose that is lipid to body condition

$$a_2 = \mu_{P_{AL}} * \varphi_2$$

Equation 2.0c First shape parameter for beta regression

$$b_2 = (1 - \mu_{P_{AL}}) * \varphi_2$$

Equation 2.0d Second shape parameter for beta regression

1.5 Developing the Equation to Estimate the Proportion of Adipose that is Protein

We used adipose composition from the dissection data to quantify a relationship between the combined proportion of protein and ash and the proportion of lipid in adipose (Fig. S3). We built a beta regression that used the combined proportion of protein and ash in adipose tissue as a response variable and the proportion of adipose that is lipid as the predictor variable (Box 1). Parameter priors are listed in Table 1. The observed percentage of ash in adipose tissue was <1% (Table S2), so we assumed the presence of ash in adipose to be negligible. Hence, the output from the fitted model is equated to the proportion of protein in adipose (P_{AP}) and can be estimated given the predicted proportion of lipid (Eqn. 3.0a-d) in our subsequent analyses.

$$P_{AP} \sim beta(a_3, b_3)$$

Equation 3.0a Beta regression fitting proportion of adipose that is protein given the proportion lipid

$$logit(\mu_{P_{AP}}) = \left(\alpha_{P_{AP}} + \beta_{P_{AP}} P_{AL} \right)$$

Equation 3.0b Logit link to linear regression relating proportion of adipose that is protein to the proportion that is lipid

$$a_3 = \mu_{P_{AP}} * \varphi_3$$

Equation 3.0c First shape parameter for beta regression

$$b_3 = (1 - \mu_{P_{AP}}) * \varphi_3$$ **Equation 3.0d** Second shape parameter for beta regression

1.6 Estimating the Proportion of Storage Muscle that is Lipid and Protein

Finally, we estimated the proportion of lipid and protein in storage muscle tissue to determine the total energy available in storage mass (Fig. 1). Given the weak relationship between the proportion of muscle that is protein (P_{UP}) and lipid (P_{UL}) in our available data (Fig. S4), we established a minimum, average, and maximum for each as observed in two studies that analyzed biceps femoris composition (Cattet, 1988; Whiteman *et al.*, 2017). Muscle protein content varies positively with feeding and activity in polar bears (Whiteman *et al.*, 2017). Samples from the Southern Beaufort Sea likely capture seasonal highs in muscle protein content given they were collected in October after bears had been active and feeding for six months (Whiteman *et al.*, 2017), while samples from Western Hudson Bay likely capture muscle protein lows given they were collected in early fall/late summer when most bears have been fasting and minimally active (Cattet, 1988). The dissection data (Cattet, 1988) were used to determine values for the proportion of muscle tissue that is lipid [minimum =0.003, average=0.0218, maximum=0.068] and combined with available literature values (Whiteman *et al.*, 2017) to determine the proportion that is protein [minimum=0.205, average=0.3738, maximum=0.62]. We assessed how energy estimates were impacted using each combination of minima and maxima (i.e., predicted energy in storage muscle assuming the proportion of lipid and protein are 1) both at the minimum observed, 2) both at the maximum observed, 3) maximum lipid and minimum protein observed and 4) minimum lipid and maximum protein observed; Table S4).

2 Developing the Scaled Mass Index

To connect polar bear body condition to underlying body composition, and thus energy stores, we must use a body condition index indicative of an individual's overall adiposity. Here, we developed a scaled mass index (*SMI*) for polar bears and compared its performance to previously proposed polar bear body condition indices, so to circumvent errors typically associated with existing methods. Existing, quantitative body condition indices are typically derived from a combination of total body mass and some morphometric measurement that indicates structure to estimate the mass that is available as energy (Cattet *et al.*, 2002; Stevenson and Woods, 2006; Rode *et al.*, 2020). Straight-line body length is a predictor of structure in ursids, reliably correlating with underlying structural components in polar bears (Cattet *et al.*, 2002). However, analyses on repeated measures of adult polar bear straight-line body length suggest that the precision around this measure is typically low (Rode *et al.*, 2020). The scaled mass index reduces errors associated with 1) the allometry of total mass and straight-line body length, both of which are indicators of structure (i.e., a larger mass can indicate larger structure as does a larger length); 2) the difference in total mass and length scales, which can lead to differences in the natural variability in each variable; and 3) defining the scaling exponent between the two interdependent variables (Peig and Green, 2009). Furthermore, *SMI* can be used across populations once the parameters are fit (Peig and Green, 2009).

We developed the equation for a polar bear *SMI* (Eqn. S3) where M_i and L_i are the mass and straight-line body length of each individual respectively, L_0 is the arithmetic mean of straight-line body length for the dataset, and b_{SMA} is the scaling exponent estimated by the standardized major axis (SMA) regression of the natural logarithm of M on the natural logarithm

of L (Peig and Green, 2009). To fit the *SMI* equation, we used a large dataset (n=723) of total mass (M), as determined from scale weights, and straight-line body length (L) that was comprised of 379 females, 342 males, and 2 bears of unknown sex spread across all age groups (Cubs: 183, Yearlings: 66)). All data were collected from the Western Hudson Bay subpopulation by Environment and Climate Change Canada (ECCC) spanning from 1966 to 2013 (Figure S1).

$$SMI_i = M_i \left[\frac{L_0}{L_i}\right]^{b_{SMA}}$$ Equation S3 (Peig and Green, 2009; their Eq. 2)

Following the steps from Peig and Green (2009), we examined the data for extreme outliers from the typical scaling pattern between total mass and straight-line body length and removed one datapoint (Figure S1) before fitting the *SMI* equation. Next, we found the best fit line on the remaining data using a standardized major axis (SMA) regression on the natural logarithm transformed data (Peig and Green, 2009) to determine the scaling exponent relating mass and length (b_{SMA}=3.16). The datapoints were standardized to the same body size using the arithmetic mean of straight-line body length from all polar bears in the remaining data (L_0=1.81m). The scaled mass index (*SMI*) can then be calculated according to Eqn. S3, which estimates the body mass each bear would be at L_0, effectively removing the variation imposed by different straight-line body lengths.

To determine how well *SMI* correlated with underlying indicators of body condition (i.e., adipose stores) in comparison to a polar bear body condition index calculated using more typical ordinary least squares regression (*OLS*) (Cattet *et al.*, 2002), we used the dissection dataset. The sum of muscle and adipose tissue was used as the best indicator of body condition given their association with energy storage (Cattet *et al.*, 2002; Peig and Green, 2009). Rather than simply

using the total masses of the tissues, which would be confounded by differences in length, we used the same procedure to determine the scaled component mass (*SCM*), an unbiased measure of composition which controls for scaling relationships and enables validation of non-destructive condition indices calculated using body mass and length (Peig and Green, 2009).

$$SCM_i = TM_i \left[\frac{L_0}{L_i}\right]^{b_{SMA_{TM}}} \text{ Equation S4}$$

TM_i is the sum of muscle and adipose tissue mass for each individual, L_i is the straight-line body length of each individual, and L_0 is again the arithmetic mean as calculated from the larger ECCC dataset. The scaling exponent ($b_{SMA_{TM}}$) is calculated from the SMA regression of the natural logarithm of tissue masses on the natural logarithm of straight-line body length (Peig and Green, 2009).

Using the fitted Eq. S3 and S4, we calculated *SMI* and *SCM* for each dissected polar bear. We found high correlation between *SMI* and the *SCM* of adipose and muscle (Pearson; r=0.968 [0.935-0.985], df=29, $p<2.2^{-16}$). The correlation is comparable to that between *SCM* and the previously calculated *OLS* index that was developed using these same dissection data (r=0.949 [0.896-0.75], df=29, $p<4.6^{-16}$) (Cattet *et al.,* 2002). As a further check, we considered the subjective fatness index values (a scale from 1-5 with 1 being the skinniest) for polar bears in the ECCC dataset (Stirling *et al.,* 2008). The subjective fatness index suggested that males were in slightly better body condition than females (Average for males: 2.94, n=160; Average for females: 2.88, n=139), although a difference is not confirmed by a Welch's t-test (t=0.579, df=266.97, p=0.563; Figure S2). Average body conditions according to the original *OLS* suggest females have a higher body condition on average than males (Average for males:79.63, Average for females: 79.7), but results were not significantly different according to Welch's t-test (t=-

0.896, *df*=254.71, p= 0.371). In contrast, the *SMI* index showed that male body condition was better than females (M: 179.7, F:167.0) with a significant difference confirmed by Welch's t-test (t=3.70, *df*=261.11, p= 2.6^{-4}). Given the advantages of *SMI* to lower impacts from measurement error on length and mass, its high correlation with the underlying energy stores, and agreement with the subjective fatness index, we used *SMI* to indicate body condition in the development of our body composition model.

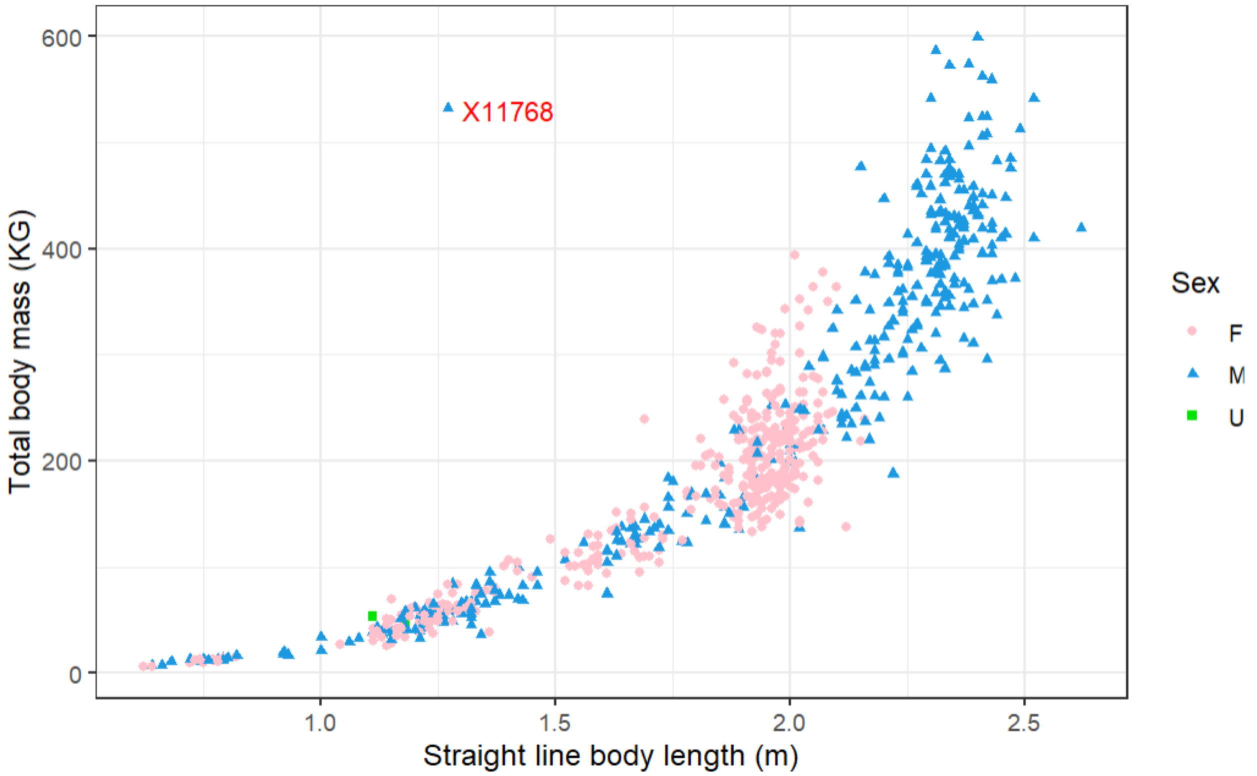

Figure S1. Total mass and straight-line body length data for 723 polar bears from the Western Hudson Bay subpopulation. Datapoints include 379 females, 342 males, and 2 bears of unknown sex spread across all age groups (Cubs: 183, Yearlings: 66). Given its large deviation from the general scaling pattern, we attributed the outlier shown in red to transcription error and removed it from calculations of the scaled mass index scaling exponent.

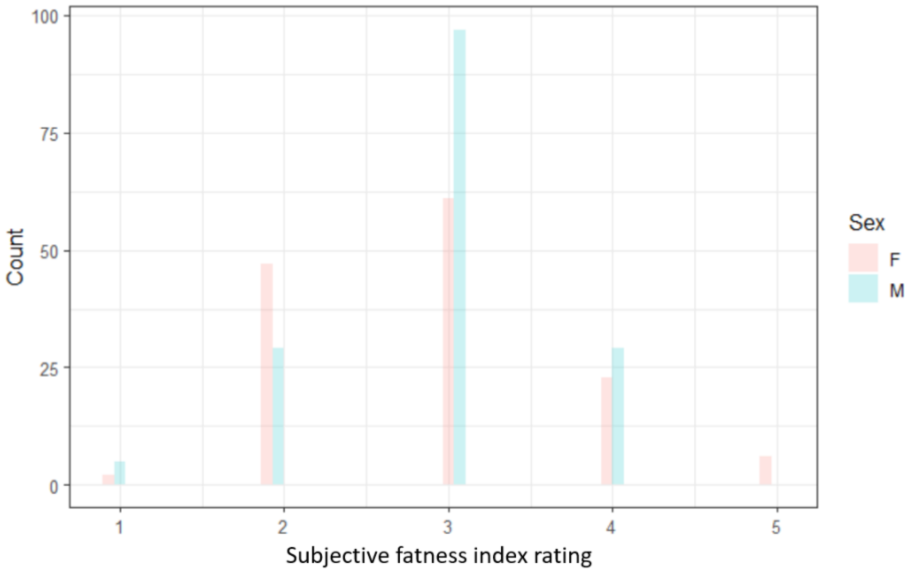

Figure S2. Histogram of subjective fatness index ratings for 299 adult polar bears from the Western Hudson Bay subpopulation. Datapoints include 139 females and 160 males.

3 Dataset Details

3.1 Dissected Bear Data

Table S1. Identification and basic characteristics of the dissected polar bears (n=31) used in this study. Bears originated from the Foxe Basin (FB), Lancaster Sound (LS), or Western Hudson Bay (WH) subpopulations. Straight-line body lengths were measured as the distance from the tip of the nose to the base of the tail when the spine is straightened, and total masses were estimated as the sum of total dissected tissues. Some assigned ages did not fall within reasonable upper/lower bounds for age-specific mass and straight-line body lengths determined from over 45 years of measurements collected in WH. Where necessary, differences in growth rate and asymptotic body length between WH and bears collected from FB (WH Females-0cm; WH Males -3cm) and LS (WH Females -4cm; WH Males -6cm) (Derocher and Stirling, 1998) were accounted for before determining that an individual's age did not match mass and/or length. Ages were re-assigned for six individuals which did not fall within reasonable upper/lower bounds; the original age is shown in brackets beside the age used here. All samples were collected in the 1980s, see Cattet (1988) for details.

Bear ID	Date of death	Sex	Age	Straight-line body length (m)	Mass (kg)	Subpopulation of origin
P13	25/10/1985	male	2	1.78	176.3	FB
P14	28/10/1985	male	3	1.68	118	FB
P15	4/11/1985	female	2	1.75	162.8	FB
P16	14/11/1985	female	13	1.8	184.6	FB
P17	19/11/1985	female	4	2.11	184	FB
P18	19/11/1985	female	0 (1)	1.22	32.4	WH
P19	18/11/1985	male	4	1.95	150.9	WH
P20	11/11/1985	male	3	1.65	117.6	WH
P21	16/10/1985	male	21	2.47	432.9	WH
P22	15/3/1986	male	11	2.27	304.1	FB
P23*	26/4/1986	female	6	1.95	157	LS
P24*	9/5/1986	male	3 (5)	1.67	114.5	LS
P25	12/5/1986	female	6	1.73	163.4	LS
P26*	23/1/1986	male	1[1]	1.24	38.6	WH
P27*	1/11/1986	male	7	2.23	335.5	WH
P28	3/11/1986	male	7	1.88	186.9	WH
P29	3/11/1986	female	1 (5)	1.52	121	WH
P30	3/11/1986	female	6	1.72	195.4	WH
P31	3/11/1986	male	8	2.03	256.3	WH
P32	4/11/1986	female	1 (4)	1.45	102.5	WH
P33	7/11/1986	male	2	1.88	166.7	WH
P34	9/11/1986	female	2	1.58	117.5	WH
P35*	8/11/1986	male	15	2.36	511.9	WH
P36	11/11/1986	female	4 (2)	1.79	116.7	WH
P37	12/11/1986	male	3 (5)	1.73	142	WH
P38	8/11/1986	male	2	1.97	142.3	WH
P39	9/11/1986	female	2	1.55	92.1	WH
P40	9/11/1986	male	12	2.3	252.7	WH
P41	10/11/1986	female	16	1.84	166.7	WH
P42	15/11/1986	male	3	1.75	158.8	WH
P43	11/11/1986	male	21	2.41	288.4	WH

*Individuals that were used to determine the chemical composition of various tissue types
[1]Note that this yearling was captured in January and thus is more similar in size to cubs of the year captured during the onshore season in WH.

Table S2. Chemical composition of polar bear muscle, viscera, adipose and bone (including brain and spinal cord) tissue. The mean, standard deviation, and ranges for the proportions of neutral lipids, protein, water, and ash in a composite sample of each tissue type (i.e., multiple samples of tissue type taken from different depots across the body and homogenised) from five polar bears (one adult female, two adult males, one subadult male, and one male cub of the year; cf. Table S1 for details) are shown. Skin was only analysed for the male cub (P26). Given that polar lipids were not measured but are present in each tissue type (Cattet *et al.*, 2001), we expect the sum of tissue components to be less than 100%.

**Tissue Type**	**Neutral Lipid (%)**	**Protein (%)**	**Water (%)**	**Ash (%)**
Muscle	5.0 ±2.00<br>*2.8-7.3*	20.9 ±0.802<br>*20.1-21.8*	70.6 ±2.35<br>*66.7-72.7*	1.12 ±0.383<br>*0.9-1.8*
Viscera	7.44 ±2.79<br>*3.2-9.7*	17.2 ±1.18<br>*15.3-18.2*	70.1 ±2.21<br>*67.9-73.2*	1.24 ±0.279<br>*1.0-1.7*
Adipose	67.1 ±11.1*<br>*54.8-78.0*	9.86 ±3.81<br>*5.8-14.0*	19.3 ± 7.99<br>*12.2-28.9*	0.360 ±0.344<br>*0.1-0.9*
Bone	17.6 ±3.61<br>*13.5-22.7*	19.0 ±2.08<br>*16.9-22.3*	43.8 ±1.05<br>*42.1-44.9*	14.1 ±1.88<br>*11.0-16.0*
Skin	20.9	29.6	42.8	3.5

***** The variation is driven by the subadult male (P24) and cub of the year (P26) with measurements of 56.6% and 54.4% respectively, while the remaining adult female and two adult males have measurements of 70.1%, 77% and 77.6% respectively.

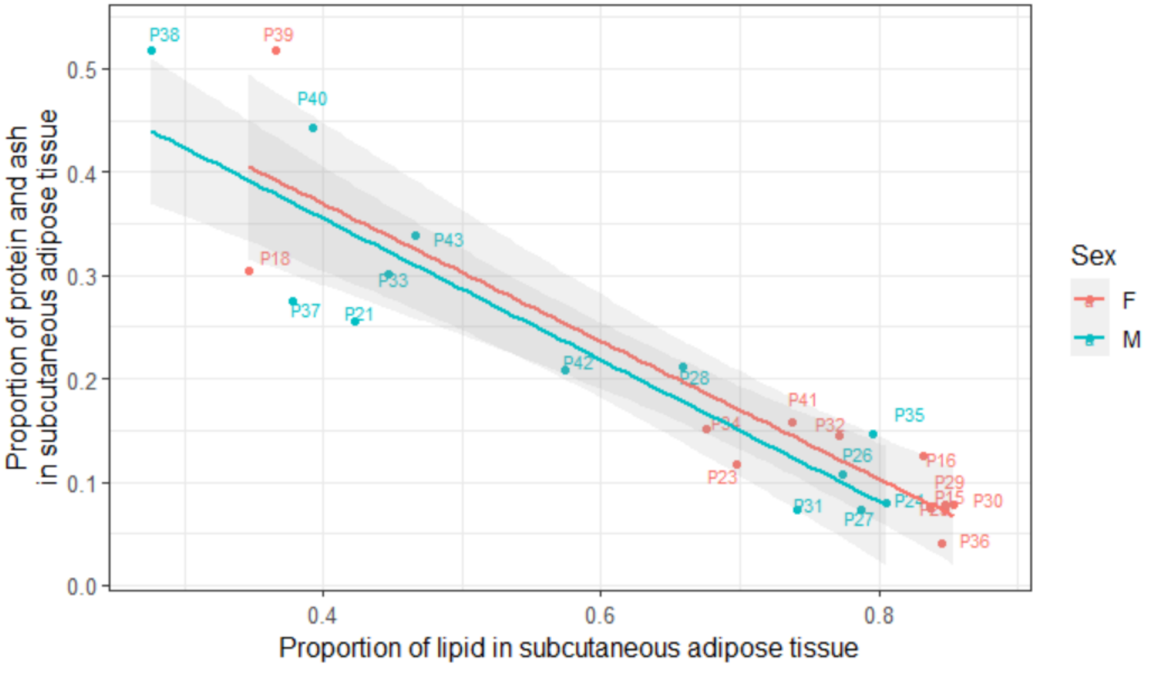

Figure S3. The proportion of lipid and of combined ash/protein in subcutaneous adipose of 25 dissected polar bears (Table S1; subcutaneous adipose samples were not analysed for P13, P14, P17, P19, P20, P22). Linear models were fit to the female (pink) and male (blue) datapoints; 95% confidence intervals are shaded in grey.

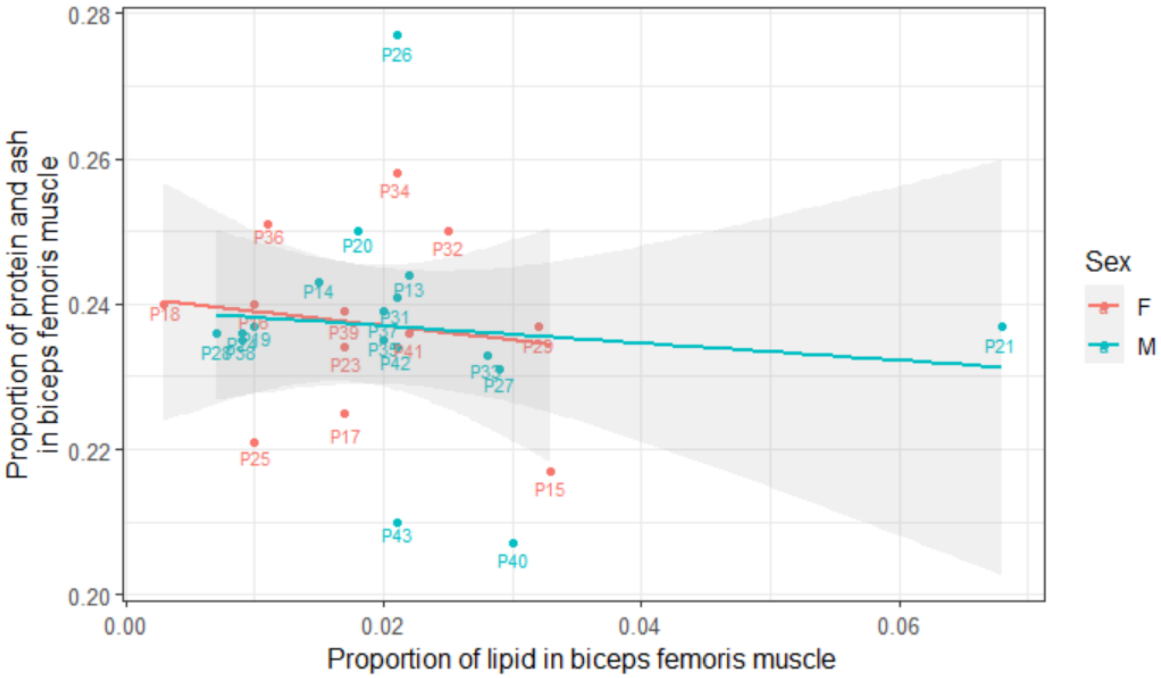

Figure S4. The percentages of lipid and combined ash/protein in biceps femoris muscle tissue of 29 dissected polar bears (Table S1; biceps femoris samples were not analysed for P30 or P22). Linear models were fit to the female (pink) and male (blue) datapoints; 95% confidence intervals are shaded in grey.

Table S3. Estimates for energy content of storage muscle tissues given minimum and maximum estimates for the proportion of muscle that is protein and lipid. Using our model-estimated masses of structural muscle (cf. Eqn. 1.0b), we determined the mass of storage muscle as the difference with total muscle (cf. Eqn. 1.2). Storage muscle was multiplied by the maximum and minimum proportion of lipid observed in the biceps femoris of our dissected bears (0.3% and 6.8%), and the maximum and minimum proportion of either the protein/ash observed in the biceps femoris of our dissected bears or protein from Whiteman et al. (2017) (20.5% and 62%), in all combinations. We assumed that stored lipid and protein contain 39.3MJ and 18.4MJ of metabolizable energy, respectively.

**Bear ID**	**Storage muscle (kg)**	**Estimated energy content of storage muscle (MJ) if:**			
		**6.8% lipid and 20.5% protein**	**6.8% lipid and 62% protein**	**0.3% lipid and 20.5% protein**	**0.3% lipid and 62% protein**
P13	50.5	325.4	711.1	196.4	582.1
P14	30.6	197.2	430.9	119.0	352.7
P15	40.8	262.9	574.5	158.7	470.3
P16	45.5	293.2	640.7	177.0	524.4
P17	41.7	268.7	587.2	162.2	480.6
P18	4.7	30.3	66.2	18.3	54.2
P19	34.6	223.0	487.2	134.6	398.8
P20	36.5	235.2	513.9	142.0	420.7
P21	88.4	569.7	1244.7	343.9	1018.9
P22	100.0	644.4	1408.0	389.0	1152.6
P23	45.0	290.0	633.6	175.0	518.7
P24	34.2	220.4	481.5	133.0	394.2
P25	49.5	319.0	697.0	192.6	570.5
P26	3.0	19.3	42.2	11.7	34.6
P27	95.1	612.9	1339.0	370.0	1096.1
P28	52.3	337.0	736.4	203.4	602.8
P29	27.5	177.2	387.2	107.0	317.0
P30	63.3	407.9	891.3	246.2	729.6
P31	77.4	498.8	1089.8	301.1	892.1
P32	27.1	174.6	381.6	105.4	312.4
P33	37.8	243.6	532.2	147.0	435.7
P34	28.7	185.0	404.1	111.6	330.8
P35	157.9	1017.6	2223.3	614.2	1819.9
P36	28.3	182.4	398.5	110.1	326.2
P37	39.6	255.2	557.6	154.0	456.4
P38	37.9	244.2	533.6	147.4	436.8
P39	20.9	134.7	294.3	81.3	240.9
P40	79.7	513.6	1122.2	310.0	918.6
P41	46.7	301.0	657.6	181.7	538.3
P42	42.1	271.3	592.8	163.8	485.2
P43	85.2	549.1	1199.7	331.4	982.0

3.2 Adipose Biopsy Data

To determine a suitable process model with which to predict the proportion of adipose that is lipid, we first explored candidate models using the "*gam*" function to fit a generalized additive model from the R-package "*mgcv*" which provides tools to analyze generalized additive (mixed) models. The adipose tissue biopsy data available for this study were collected solely from Western Hudson Bay during the summer period. Despite the seasonal influence on lipid content in adipose (Ramsay *et al.*, 1992; Thiemann *et al.*, 2006; McKinney *et al.*, 2014; Sciullo *et al.*, 2016), our dataset includes extremely low (<30%) and high values (>90%), encompassing the range of potential values observed across different subpopulations, cohorts, and seasons (Thiemann *et al.*, 2006). Here, we seek to link adipose lipid content to physiological factors beyond the natural seasonality of food availability, focusing on body condition, sex, and age as potential drivers. Random effects across different individuals were not significant ($p>0.5$ for all candidate models). Sex was the most significant predictor ($p<0.001$ for all candidate models); age class was only significant for cubs ($p<0.01$) (i.e., yearlings, subadults and adults were not significantly different from each other after sex was accounted for) and only if two male cub outliers whose lipid values were much lower than their lengths and masses would have suggested are included in the analysis (exclusion results in $p > 0.2$); and body condition (*SMI*) was only significant in candidate models where sex was included ($p \leq 0.012$). Although *SMI* and sex were significant variables, they only explained a small amount of the variation in the proportion of adipose that is lipid ($R^2_{adj} = 0.14$, Deviance explained = 15.5%).

Table S4. Model selection and multi-model inference based on Akaike's Information Criterion modified for small sample sizes (AICc), applied to the beta regression that predicts the proportion of lipid in subcutaneous adipose tissue biopsies for A) all sampled polar bears, and B) with two outliers removed. The removed outliers were two male cubs of the year that had extremely low values for the proportion of lipid in the adipose biopsy given their lengths and masses. Model comparisons were performed using the R package "*AICcmodavg*". The number of parameters for each model (K) and the difference between each model's AICc and the best fit model (ΔAIC_C) are given.

A)

**Model**	**K**	**AICc**	**ΔAICc**
Class, sex and SMI	7	-284.48	0.00
Class and sex	6	-277.31	7.16
SMI and sex	4	-274.97	9.51
Sex only	3	-270.91	13.57
Class only	5	-256.86	27.62
SMI only	3	-255.71	28.77

B)

**Model**	**K**	**AICc**	**ΔAICc**
SMI and sex	4	-306.99	0.00
Sex only	3	-304.12	2.87
Class, sex and SMI	7	-303.93	3.06
Class and sex	6	-299.57	7.42
SMI only	3	-288.81	18.18
Class only	5	-283.94	23.05

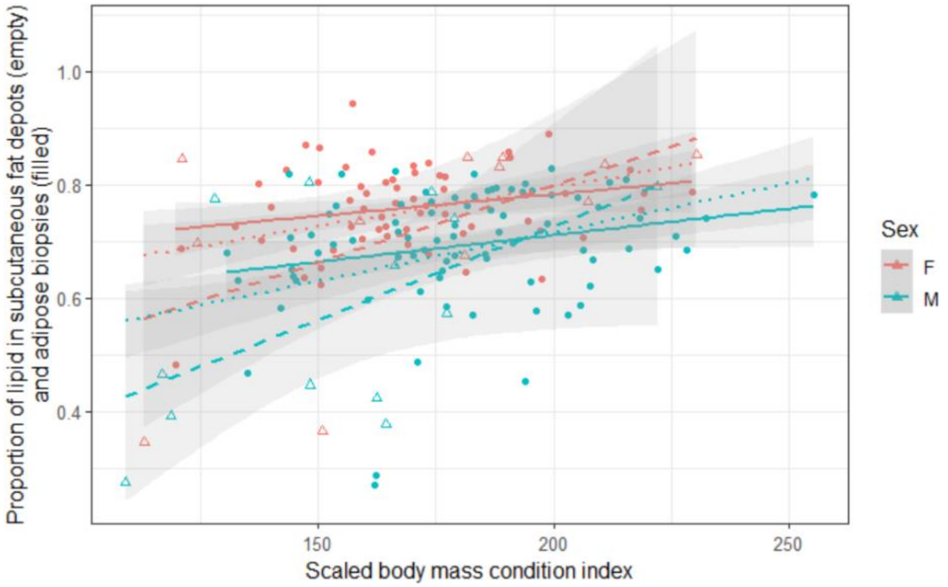

Figure S5. The percent of lipid in subcutaneous adipose tissue biopsies (filled circles) and dissected subcutaneous adipose depots (empty triangles). Linear models were fit to the biopsy data (solid lines), the dissection data (dashed lines), and the combined data (dotted lines) for males (blue) and females (pink); 95% confidence intervals are shaded in grey.

4 Priors and Model Metrics

We set weakly informative priors on the intercepts and slope parameters for all regressions (Eqn. 1.0a, 1.1, 1.4, 2.0, 3.0), drawing from normal distributions with mean 0 and a standard deviation of 2 (Table 1), so that a change of one standard deviation in the predictor variables is unlikely to illicit a more than one standard deviation in the outcome variable. The standard deviations around logged mass of total muscle (Eqn. 1.0a) and logged mass of structure non-muscle (Eqn. 1.1) were drawn from a uniform distribution, restricted to positive values below 1. The precision for the proportion of storage that is muscle (Eqn. 1.4) was restricted to positive values under 100. STAN numerically calculates the joint density function during the fitting process, moving in the entire space of all parameters, and unlike graphical models does not require proper priors to form proper posteriors (Nishio and Arakawa, 2019). Since we did not have prior expectations on the values for the beta regression parameters (1.4b), we also fit the models (Box 1) with flat priors on all parameters (Table 1) so as not to interfere with the sampler and to evaluate the strength of the priors' influence. For all model fittings (Box 1), we used a conservative tuning period of 50,000 for four chains with 50,000 samples.

Table S5. Convergence and efficiency diagnostics for model fits of the combined model - which gives total muscle (Eqn. 1.0a), structure non-muscle (Eqn. 1.1) and the proportion of storage that is muscle (Eqn. 1.4) given three different structures in Eqn. 1.4 (no difference in model parameters between sexes, intercept allowed to vary by sex, slope allowed to vary by sex) – and models that estimate the proportion of adipose that is lipid and protein. The potential scale reduction factor ($\hat{R}$) convergence diagnostic, which compares the between- and within-chain estimates for model parameters and other univariate quantities of interest, is shown. Values less than 1.1 for each parameter indicate that chains have mixed well (i.e., the between- and within-chain estimates agree). The effective sample size (ESS) is a useful measure for sampling efficiency, measuring the amount by which autocorrelation in samples increases uncertainty (standard errors) relative to an independent sample. Parameters with an effective sample size less than 10% of the total sample size are acceptable. We tracked each iteration for divergence (which indicates when the sampler is not drawing samples from the entire posterior distribution and inferences will be biased), and saturation of max tree-depth (which can indicate unidentifiability issues). We also considered the energy Bayesian fraction of missing information (E-BFMI), which quantifies the efficacy of the momentum resampling in between Hamiltonian trajectories. A value below 0.2 indicates the model may need to be re-parameterized. See the STAN model workflow presented by Betancourt (2017) for details. Note that we only show results from models fit with weakly informative priors but there was not a significant difference in diagnostics when models were fit with unrestricted priors (Table S6-S8).

Model	Structure	$\hat{R}$ >1.1	ESS >10%N	Iterations with divergence	Iterations with max tree depth	E-BFMI >0.2
Total muscle (Eq. 1.0a), structure non-muscle (Eq. 1.1), proportion of storage that is muscle (Eq. 1.4)	No difference between sexes	None	None	0	0	Yes
	Intercept varies by sex	None	Intercept hyperparameters	49.7%	0	Yes
	Slope varies by sex	α_{lU_0}	Only β_{lKnU_1} is acceptable	30.0%*	5.92%	Yes
Proportion of adipose that is lipid (Eq. 2.0a-d)	Base	None	None	0	0	Yes
Proportion of adipose that is protein (Eq. 3.0a-d)	Base	None	None	0	0	Yes

*>80% of divergences occurred in a single chain

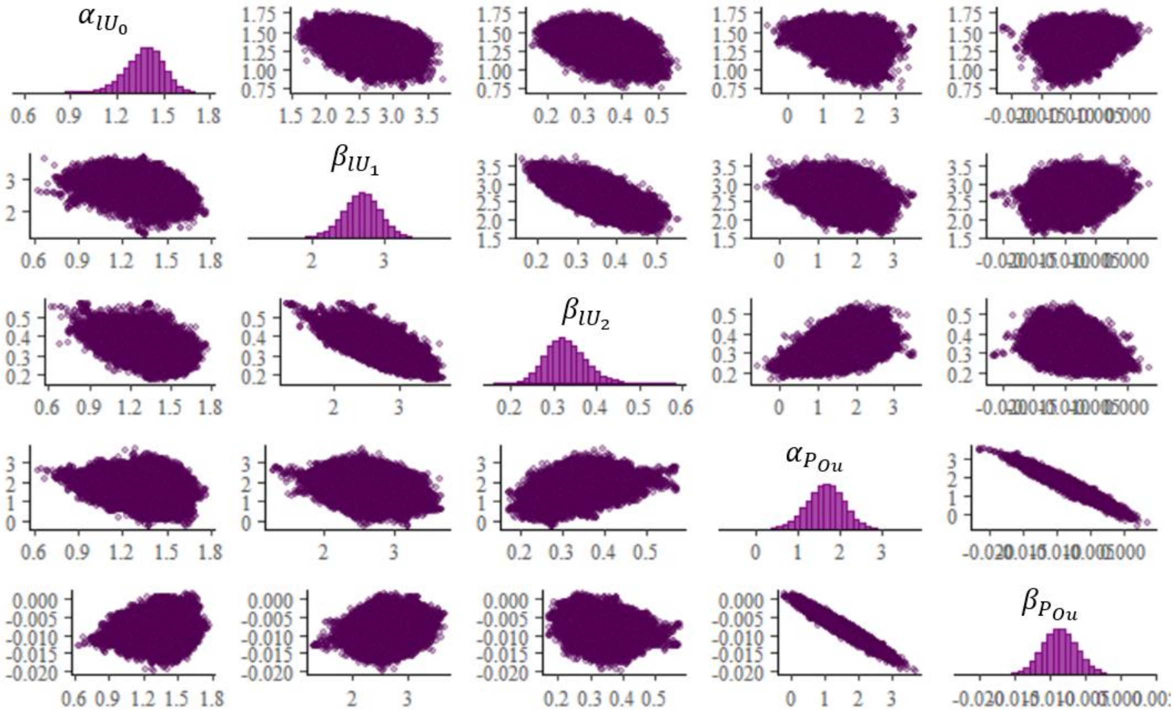

Figure S6. Pairs correlation plot for parameters estimated by the base combined model fitting process, in which logged total muscle (*U*) (Eq. 1.0a) and proportion of storage that is muscle (P_{O_U}) (Eqn. 1.4) are both fit using weakly informative priors (Table 1). Given that the "observed" proportion of storage that is muscle used to fit equation 1.4 was calculated using the latent variable of structural muscle, which in turn was calculated from the fitted equation 1.0a, we must check for covariance between the parameter estimates from equation 1.0a (α_{lU_0}, β_{lU_1}, β_{lU_2}) and the parameter estimates from equation 1.4b ($\alpha_{P_{Ou}}$, $\beta_{P_{Ou}}$). The diagonal shows a histogram of estimates for each parameter while the off diagonals show the pairs plot for each combination. The estimates for each iteration of chain 1 and 2 are shown below the diagonal while the estimates for each iteration of chain 3 and 4 are shown above the diagonal.

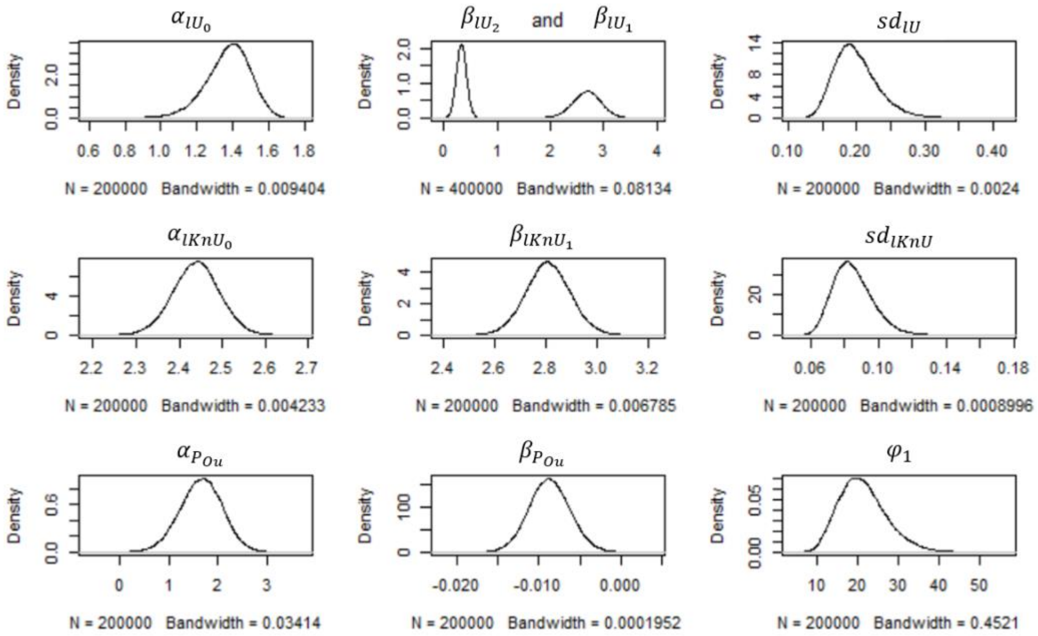

Figure S7. Density plots for parameter estimates from the base combined fitting of logged total muscle (*U*), logged structure non-muscle (K_{nU}), and proportion of storage that is muscle (P_{O_U}) given weakly informative priors (Table 1). The first row shows parameters from the logged total muscle equation (Eqn. 1.0a); the second row shows parameter estimates from the logged total structure non-muscle equation (Eqn. 1.1); and the third row shows parameter estimates from the proportion of storage that is muscle equation (Eqn. 1.4). The density plots are combined across all iterations and chains.

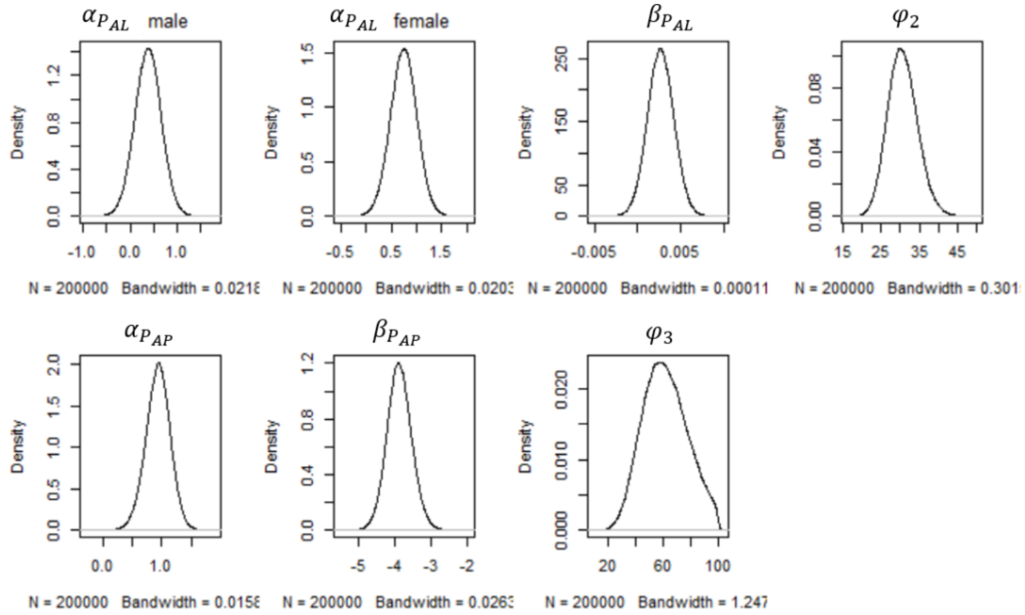

Figure S8. Density plots for parameter estimates from the fitting of the equations to estimate the proportion of adipose that is lipid (P_{AL}) from body condition and the proportion of adipose that is protein (P_{AP}) from the estimated lipid content given weakly informative priors (Table 1). The first row shows parameters from the equation to estimate the proportion of adipose that is lipid (Eqn. 2.0); the second row shows parameter estimates from the equation to estimate the proportion of adipose that is protein content given lipid (Eqn. 3.0). The density plots are combined across all iterations and chains.

5 Parameter Estimates

Table S6. Mean and standard deviation of all parameters estimated during the combined model fitting process for logged total muscle mass (U; Eqn. 1.0a), logged mass of structure non-muscle (K_{nU}; Eqn. 1.0b), and the proportion of storage that is muscle (P_{O_U}; Eqn. 1.4) (Box 1 top panel). The model was fitted using weakly informative priors from a uniform distribution and from a normal distribution (Table 1).

Parameter	Description	Mean ± standard deviation	
		**Uniform**	**Normal**
	Equation 1.0a		
α_{lU_0}	Intercept	1.36±0.125	1.37±0.124
β_{lU_1}	Slope from logged length	2.72±0.256	2.69±0.256
β_{lU_2}	Slope from logged adipose	0.324±0.0474	0.327±0.0478
sd_{lU}	Standard deviation around logged total muscle mass	0.200±0.0324	0.199±0.0325
	Equation 1.1		
α_{lKnU_0}	Intercept	2.44±0.0523	2.44±0.0561
β_{lKnU_1}	Slope contribution from logged straight-line body length	2.81±0.0903	2.81±0.0901
$sd_{lK_{nU}}$	Standard deviation around logged total structure non-muscle mass	0.0851±0.0119	0.0851±0.0118
	Equation 1.4		
$\alpha_{P_{Ou}}$	Intercept	1.71±0.444	1.66±0.436
$\beta_{P_{Ou}}$	Slope contribution from body condition (*SMI*)	-0.00910±0.00254	-0.00874±0.00251
φ_1	Variance around the proportion of storage that is muscle	21.4±5.90	21.3±5.88

Table S7. Mean and standard deviation of all parameters estimated during the model fitting process for the proportion of adipose that is lipid (P_{AL}; Eqn. 2.0) (Box 1 bottom left panel) using all data except two outlier male cubs of the year (Fig. 3B). All candidate models were fit using weakly informative priors from a uniform distribution and from a normal distribution (Table 1).

**Parameter**	**Description**	**Mean ± standard deviation**	
		**Uniform**	**Normal**
Equation 2.0			
$\alpha_{P_{AL[M]}}$	Male specific intercept	0.407±0.256	0.378±0.279
$\alpha_{P_{AL[F]}}$	Female specific intercept	0.747±0.241	0.745±0.260
$\beta_{P_{AL}}$	Slope contribution from body condition (*SMI*)	0.00252±0.00140	0.00265±0.00152
φ_2	Variance around the proportion of adipose that is lipid	32.8±3.92	30.6±3.85

Table S8. Mean and standard deviation of all parameters estimated during the model fitting process for the proportion of adipose that is protein (P_{AP}; Eqn. 3.0) (Box 1 bottom right panel). All candidate models were fit using weakly informative priors from a uniform distribution and from a normal distribution (Table 1).

**Parameter**	**Description**	**Mean ± standard deviation**	
		**Uniform**	**Normal**
Equation 3.0			
$\alpha_{P_{AP}}$	Intercept	1.01±0.204	0.934±0.206
$\beta_{P_{AP}}$	Slope contribution from the proportion of adipose that is lipid	-4.01±0.342	-3.88±0.343
φ_3	Variance around the proportion of adipose that is protein	64.3±18.1	61.6±15.9

6 Model Testing

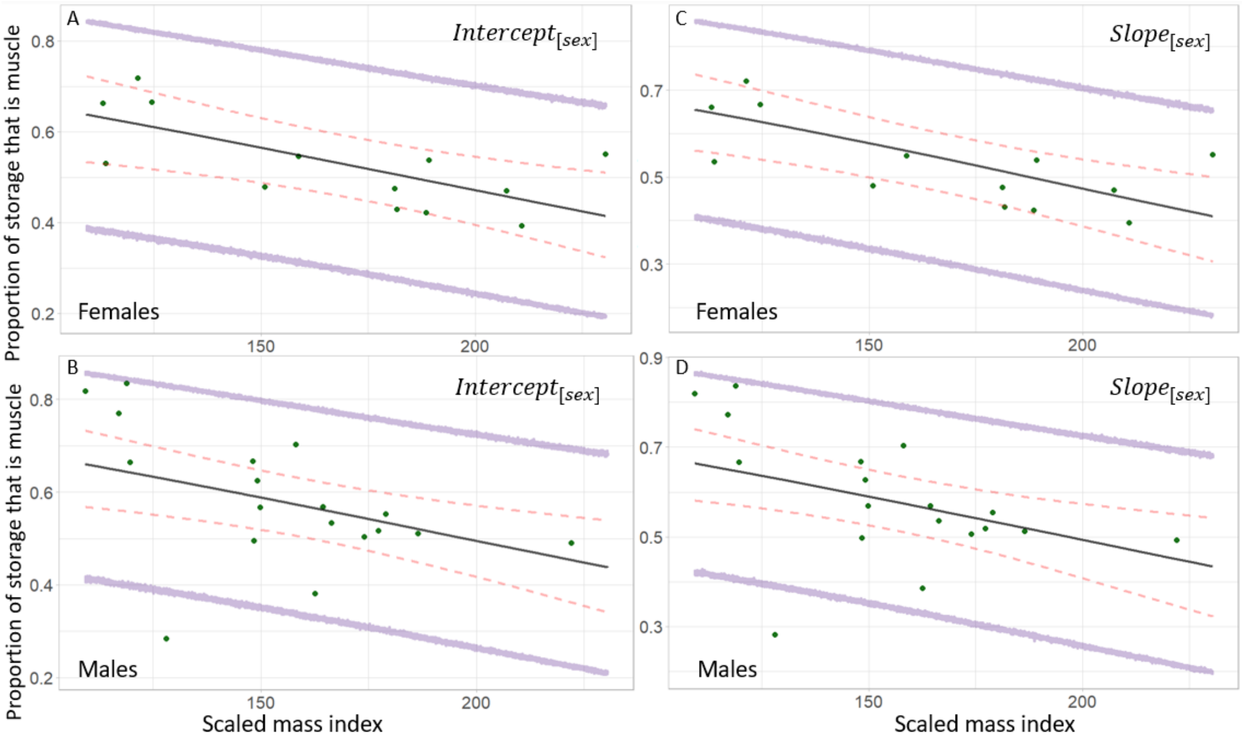

Figure S9. Predictions for the proportion of storage that is muscle (P_{O_U}) given the fitted combined model process across a range of body conditions (scaled mass index) where either the intercept (Eqn. S3a) (A, B) or slope (Eqn. S3b) (C, D) is allowed to vary by sex. The beta regression predicted proportion of storage that is muscle (black line), that was fit using the model predicted latent variable mass of structural muscle (green circles) and weakly informative priors, is shown for females (A, C) and males (B, D). The credible intervals (red dashed) show the prediction error associated with parameter estimate uncertainties, and the error associated with the data model variance is shown by the prediction intervals (purple). Note that in panels (C) and (D), both the estimated latent variable for the proportion of storage that is muscle (Eqn. 1.3; green circles), and the proportion of storage that is muscle predicted via the fitted beta regression (Eqn. 1.4; black line) are negatively correlated with scaled mass index.

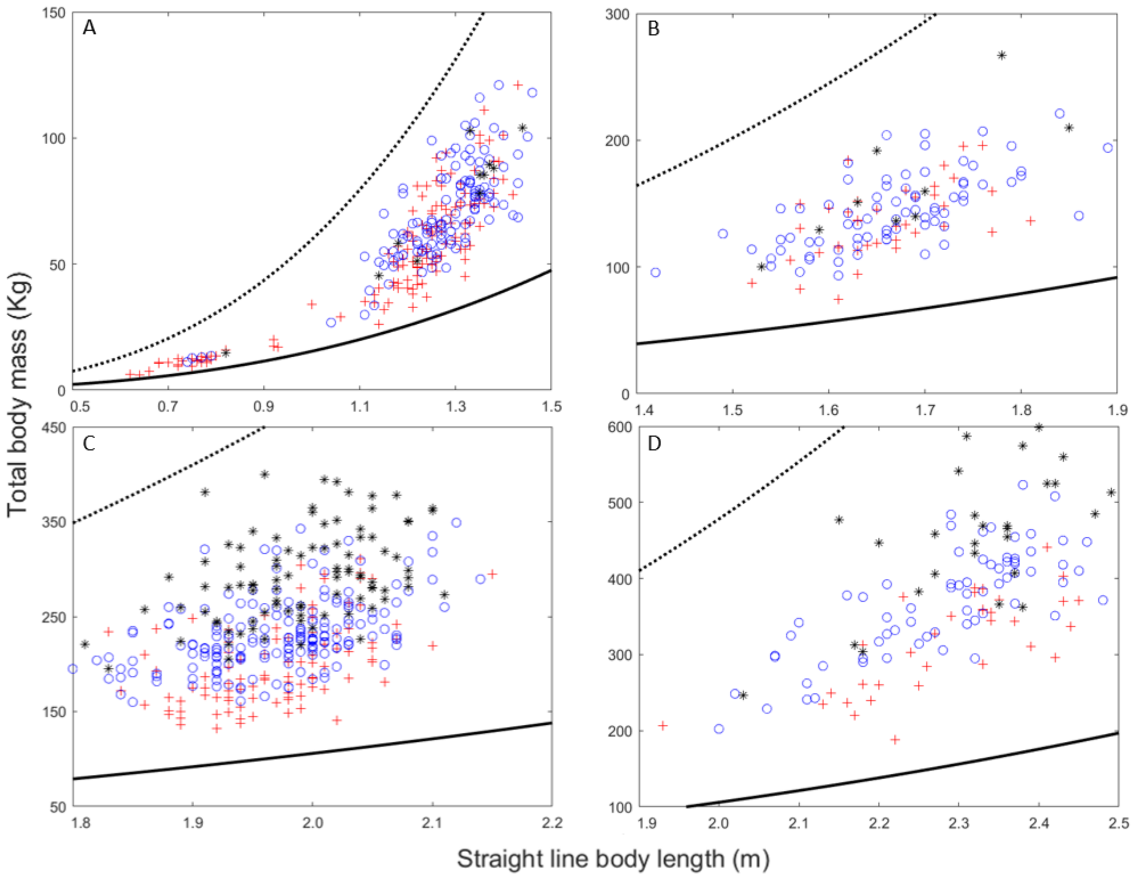

Figure S10. Straight-line body lengths and total body masses of polar bears in Western and Southern Hudson Bay for (A) cubs of the year (B) yearlings (C) subadult and adult females and (D) subadult and adult males, with data from Molnár et al (2009; their figure 4). Red crosses are bears classified as ‘1’ or ‘2’ on a subjective fatness scale (Stirling *et al.*, 2008), open blue circles are bears classified as ‘3’, black asterisks are bears classified as ‘4’ or ‘5’. Solid lines show predicted structural mass from the multi-storage model as a function of straight-line body length, dotted lines show an approximate upper bound to total body mass, taken as four times the structural mass as calculated via the single-storage model.

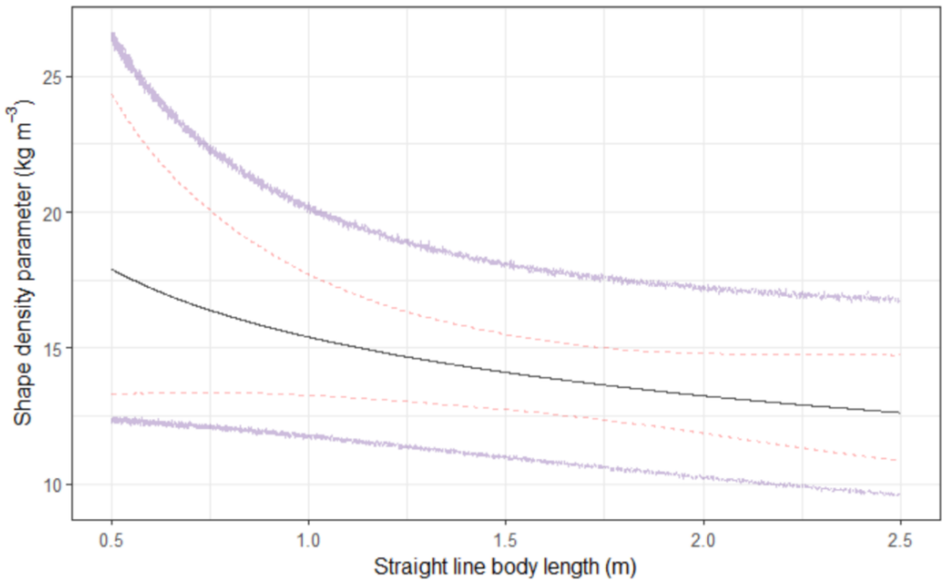

Figure S11. Straight-line body lengths and associated shape-density parameters to calculate structural mass. The shape-density parameter relates the cubed length of a polar bear to their structural mass. Using the structural mass estimated by the multi-storage model (Fig. S10), we back-calculated the shape-density value for each length as $\rho_{STR}k = \frac{M_{STR}}{L^3}$. The credible intervals (red dashed lines) show the prediction error associated with parameter estimate uncertainties. The error associated with the data model variance is shown by the prediction intervals (purple) and increases towards the smaller straight-line body lengths where data are lacking.

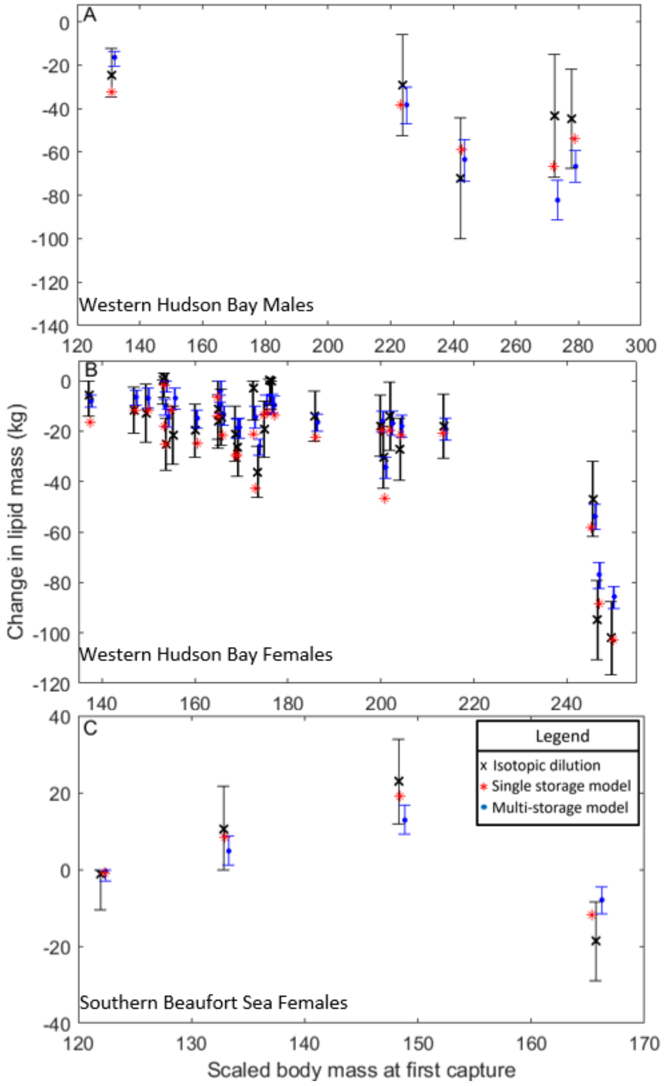

Figure S12. Predictions for the change in lipid mass between repeated measurements of adult polar bears according to the isotopic dilution model (black crosses), the single-storage compartment body composition model (red stars), and the multi-storage compartment body composition model developed here, using the lower bounds of the beta regression parameters used to calculate the proportion of adipose that is lipid (Eqn. 2.0) (blue circles). Panel (A) shows predictions for adult males in the Western Hudson Bay (WH) subpopulation, panel (B) shows predictions for adult females in the WH subpopulation, and panel (C) shows predictions for adult females in the Southern Beaufort Sea (SB) subpopulation. The error bars associated with isotopic dilution measurements show the 2.7% error associated with isotopic dilution measurements in bears (Atkinson and Ramsay, 1995). The estimates for the multi-storage body composition model were calculated using parameter values from the base model structures (Box 1) and weakly informative priors. The error bars on the multi-storage body composition model values were calculated using the maximum and minimum values for protein and associated lipid content in muscle tissue. Isotopic dilution measures are located along the exact scaled body mass measures, while the estimates from both body composition models are offset to increase readability. Note that the single-storage body composition model was trained using these specific male WH bears and the majority of WH females. The RMSE values when comparing isotopic dilution changes in lipid mass to single-storage and multi-storage model estimates were 9.2 and 11.1 respectively, 13.7 and 21.0 when only considering WH males, 4.0 and 7.9 when only considering SB females, and 8.7 and 8.6 when only considering WH females.

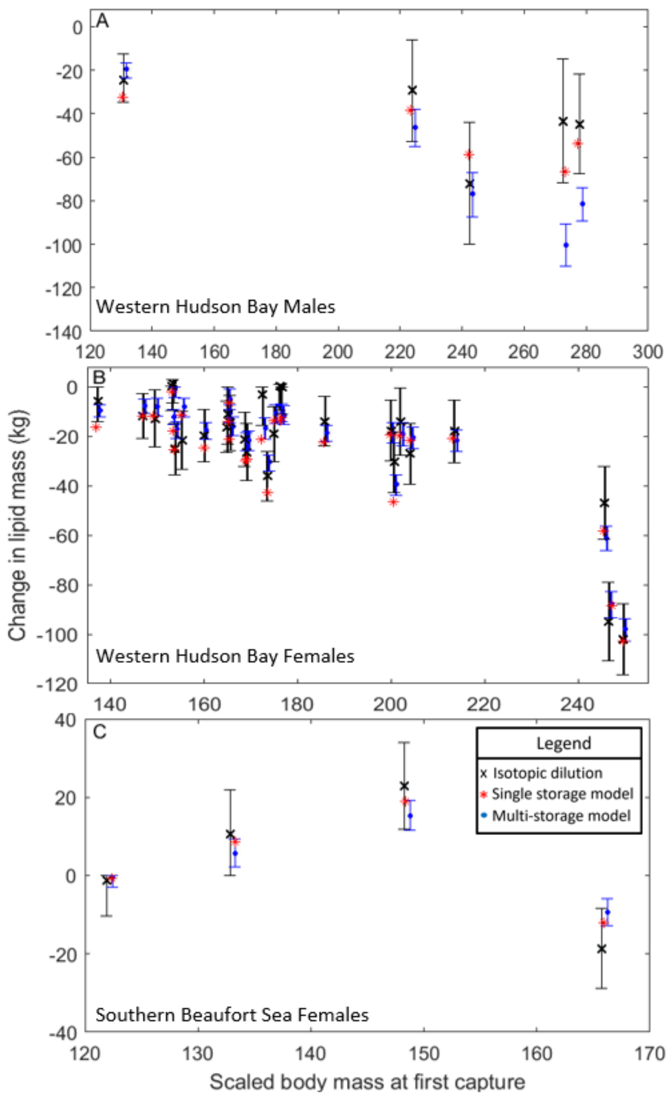

Figure S13. Predictions for the change in lipid mass between repeated measurements for adult polar bears according to the isotopic dilution model (black crosses), the single-storage compartment body composition model (red stars), and the multi-storage compartment body composition model with the intercept relating scaled mass index to the proportion of storage that is muscle allowed to vary by sex (Eqn. S1a, S2a) (blue circles). Panel (A) shows predictions for adult males in the Western Hudson Bay (WH) subpopulation, panel (B) shows predictions for adult females in the WH subpopulation, and panel (C) shows predictions for adult females in the Southern Beaufort Sea (SB) subpopulation. The error bars associated with isotopic dilution measurements show the 2.7% error associated with isotopic dilution measurements in bears (Atkinson and Ramsay, 1995). The estimates for the multi-storage body composition model are calculated using parameter values fit from weakly informative priors. The error bars on the multi-storage body composition model values were calculated using the maximum and minimum values for protein and associated lipid content in muscle tissue. Isotopic dilution measures are located along the exact scaled body mass measures, while the estimates from both body composition models are offset to increase readability. Note that the single-storage body composition model was trained using these specific male WH bears and the majority of WH females. The RMSE values when comparing isotopic dilution changes in lipid mass to single-storage and multi-storage model estimates were 9.2 and 13.0 respectively, 13.7 and 29.4 when only considering WH males, 4.0 and 6.7 when only considering SB females, and 8.7 and 7.6 when only considering WH females.

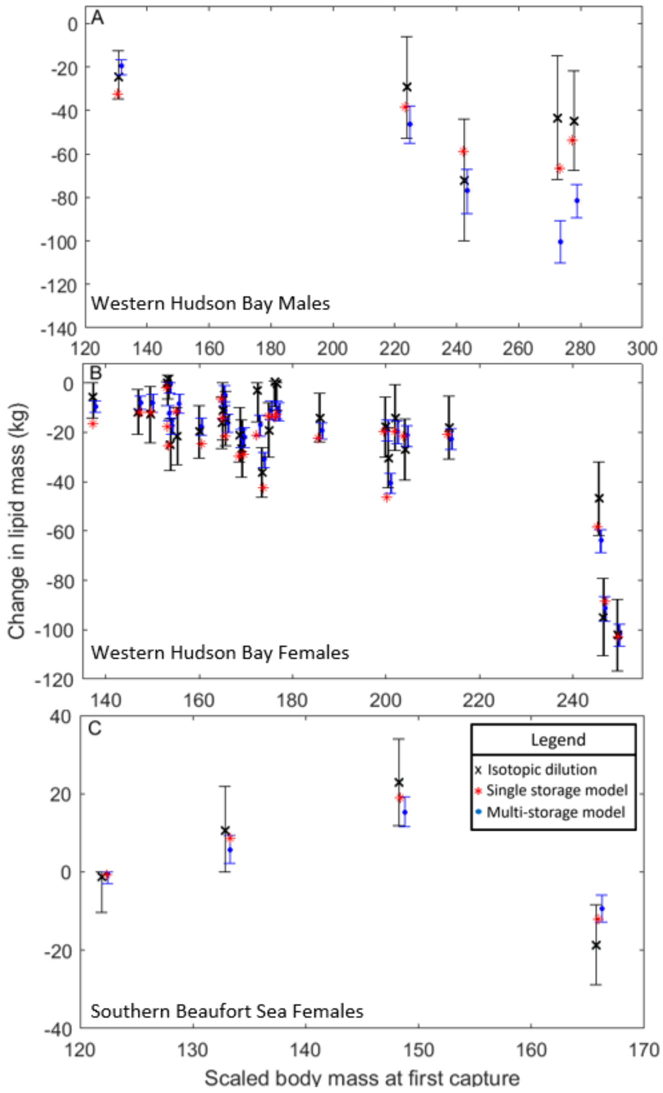

Figure S14. Predictions for the change in lipid mass between repeated measurements for adult polar bears according to the isotopic dilution model (black crosses), the single-storage compartment body composition model (red stars), and the multi-storage compartment body composition model with the slope relating scaled mass index to the proportion of storage that is muscle allowed to vary by sex (Eqn. S1b, S2b) (blue circles). Panel (A) shows predictions for adult males in the Western Hudson Bay (WH) subpopulation, panel (B) shows predictions for adult females in the WH subpopulation, and panel (C) shows predictions for adult females in the Southern Beaufort Sea (SB) subpopulation. The error bars associated with isotopic dilution measurements show the 2.7% error associated with isotopic dilution measurements in bears (Atkinson and Ramsay, 1995). The estimates for the multi-storage body composition model are calculated using parameter values fit from weakly informative priors. The error bars on the multi-storage body composition model values were calculated using the maximum and minimum values for protein and associated lipid content in muscle tissue. Isotopic dilution measures are located along the exact scaled body mass measures, while the estimates from both body composition models are offset to increase readability. Note that the single-storage body composition model was trained using these specific male WH bears and the majority of WH females. The RMSE values when comparing isotopic dilution changes in lipid mass to single-storage and multi-storage model estimates were 9.2 and 13.7 respectively, 13.7 and 31.4 when only considering WH males, 4.0 and 6.5 when only considering SB females, and 8.7 and 7.7 when only considering WH females.

7 Implementing the Multi-Storage Model

Here we provide an example of how storage muscle mass and adipose mass, as well as subsequent lipid and protein mass within storage, can be calculated from the total mass and straight-line body length of a polar bear (Px). The total mass of this hypothetical polar bear is assumed to be 250KG with a straight-line body length of 1.8m and we use multi-storage model parameters that were estimated with normally distributed priors (Table S6). First, we calculate the bear's body condition according to the scaled mass index and subsequently estimate the proportion of storage that is muscle (Eqn. 1.4) as follows:

$$SMI_i = M_i \left[\frac{L_0}{L_i}\right]^{b_{SMA}}$$

$$SMI_{Px} = 250 \left[\frac{1.81}{1.8}\right]^{3.16}$$

$$SMI_{Px} = 255$$

$$P_{O_U} = \frac{exp(\alpha_{P_{Ou}} + \beta_{P_{Ou}} SMI)}{(1 + exp(\alpha_{P_{Ou}} + \beta_{P_{Ou}} SMI))}$$

$$P_{O_{U_{Px}}} = \frac{exp(1.66 + (-0.00874) \times 255)}{(1 + exp(1.66 + (-0.00874) \times 255))}$$

$$P_{O_{U_{Px}}} = 0.36$$

Next, we estimate the amount of structural muscle (K_U; Eqn. 1.0b) and structure non-muscle (K_{nU}; Eqn. 1.1) and sum to give an estimate for the mass of structure (K; Eqn. 4.0 in the main text). Using the measured value of total body mass (M) and our estimate of structural mass, we can then estimate storage mass (O) as the difference between the two (Eqn. 4.1 in the main text).

$$K_U = exp(\alpha_{lU_0} + \beta_{lU_1} \log(L))$$

$$K_{U_{Px}} = exp(1.37 + 2.69 \log(1.8))$$

$$K_{U_{Px}} = 19.1 \text{ kg}$$

$$K_{nU} = exp(\alpha_{lKnU_0} + \beta_{lKnU_1} \log(L))$$

$$K_{nU_{Px}} = exp(2.44 + 2.81 \log(1.8))$$

$$K_{nU_{Px}} = 59.8 \text{ kg}$$

$K = K_U + K_{nU}$

$K_{Px} = 19.1 + 59.8$

$K_{Px} = 78.9$ kg

$O = M - K$

$O_{Px} = 250 - 78.9$

$O_{Px} = 171.1$ kg

Using the calculated mass of storage and proportion of storage that is muscle we estimate the mass of storage muscle (O_U; Eqn. 4.2a in the main text) and the mass of storage adipose (A; Eqn. 4.2b in the main text).

$O_U = O * P_{O_U}$

$O_{U_{Px}} = 171.1 * 0.36$

$O_{U_{Px}} = 61.6$ kg

$A = O * (1 - P_{O_U})$

$A_{Px} = 171.1 * (1 - 0.36)$

$A_{Px} = 109.5$ kg

Using the *SMI* value, the proportion of adipose that is lipid (Eqn. 2.0) and the subsequent proportion that is protein (Eqn. 3.0) are estimated as follows:

$$P_{AL} = \frac{exp(\alpha_{P_{AL_{[sex]}}} + \beta_{P_{AL}} SMI)}{(1 + exp(\alpha_{P_{AL_{[sex]}}} + \beta_{P_{AL}} SMI))}$$

$$P_{AL_{Px}} = \frac{exp(0.745 + 0.00265 \times 255)}{(1 + exp(0.745 + 0.00265 \times 255))}$$

$$P_{AL_{Px}} = 0.805$$

$$P_{AP} = \frac{exp(\alpha_{P_{AP}} + \beta_{P_{AP}} P_{AL})}{(1 + exp(\alpha_{P_{AP}} + \beta_{P_{AP}} P_{AL}))}$$

$$P_{AP_{Px}} = \frac{exp(0.934 + (-3.88 \times 0.805))}{(1 + exp(0.934 + (-3.88 \times 0.805)))}$$

$$P_{AP_{Px}} = 0.10$$

The minimum, average, or maximum observed values of the proportion of storage muscle that is lipid (P_{UL}; [minimum =0.003, average = 0.0218, maximum=0.068]) and protein (P_{UP}; [minimum=0.205, average=0.3738, maximum=0.62]) can be used to determine the mass of lipids and protein in storage muscle. Users might consider the activity levels and food deprivation the bear has been experiencing in recent months to help in this selection (Whiteman *et al.,* 2017). Here we assume our female bear has been fasting on shore with minimal movement for a month, leading us to select the average values. The total mass of storage lipids (O_L; Eqn. 5.0 in main

text) and storage proteins (O_L; Eqn. 5.1 in main text) can then be estimated and converted into total storage energy (E_O; Eqn. 5.2 in main text) using their metabolizable energy content (lipid: $\varepsilon_L = 39.3\ \frac{MJ}{KG}$, and protein: $\varepsilon_P = 18.4\ \frac{MJ}{KG}$) (Elia and Cummings, 2007).

$$O_L = P_{AL}A + P_{UL}O_U$$
$$O_{L_{Px}} = 0.805 \times 109.5 + 0.0218 \times 61.6$$
$$O_{L_{Px}} = 89.5\ \text{kg}$$

$$O_P = P_{AP}A + P_{UP}O_U$$
$$O_{P_{Px}} = 0.101 \times 109.5 + 0.3738 \times 61.6$$
$$O_{P_{Px}} = 34.1\ \text{kg}$$

$$E_O = \varepsilon_L O_L + \varepsilon_P O_P$$
$$E_{O_{Px}} = 39.3 \times 89.5 + 18.4 \times 34.1$$
$$E_{O_{Px}} = 4145\ \text{MJ}$$

8 References

Atkinson SN, Ramsay MA (1995) The Effects of Prolonged Fasting of the Body Composition and Reproductive Success of Female Polar Bears (*Ursus maritimus*). *Funct Ecol* 9: 559.

Betancourt M (2017) Robust Statistical Workflow with RStan. https://betanalpha.github.io/assets/case_studies/rstan_workflow.html?fbclid=IwAR3v8LattWabJp8K8UgjLB3FZ4QqMoHNEoOUlPvQMhtwkdO_bR3Wip_TVSk (last accessed 29 September 2021).

Branscum AJ, Johnson WO, Thurmond MC (2007) Bayesian beta regression: Applications to household expenditure data and genetic distance between foot-and-mouth disease viruses. *Aust N Z J Stat* 49: 287–301.

Cattet M (1988) Aspects of Physical Condition in Black Bears and Polar Bears. University of Alberta.

Cattet MRL, Caulkett NA, Obbard ME, Stenhouse GB (2002) A body-condition index for ursids. *Can J Zool* 80: 1156–1161.

Cattet MRL, Watts PD, Sim JS (2001) Variation in the chemical composition of adipose tissue of three species of ursids. *Can J Zool* 79: 1512–1517.

Derocher A, Stirling I (1998) Geographic variation in growth of polar bears (*Ursus maritimus*). *J Zool* 245: 65–72.

Elia M, Cummings JH (2007) Physiological aspects of energy metabolism and gastrointestinal effects of carbohydrates. *Eur J Clin Nutr* 40–74.

Gelman A, Hill J (2006) Data Analysis Using Regression and Multilevel/Hierarchical Models. Cambridge University Press, Cambridge.

McKinney MA, Atwood T, Dietz R, Sonne C, Iverson SJ, Peacock E (2014) Validation of adipose lipid content as a body condition index for polar bears. *Ecol Evol* 4: 516–527.

Molnár PK, Klanjscek T, Derocher AE, Obbard ME, Lewis MA (2009) A body composition model to estimate mammalian energy stores and metabolic rates from body mass and body length, with application to polar bears. *J Exp Biol* 212: 2313–2323.

Nishio M, Arakawa A (2019) Performance of Hamiltonian Monte Carlo and No-U-Turn Sampler for estimating genetic parameters and breeding values. *Genet Sel Evol* 51: 73–12.

Peig J, Green AJ (2009) New perspectives for estimating body condition from mass/length data: the scaled mass index as an alternative method. *Oikos* 118: 1883–1891.

Ramsay MA, Mattacks CA, Pond CM (1992) Seasonal and sex differences in the structure and chemical composition of adipose tissue in wild polar bears (*Ursus maritimus*). *J Zool* 228: 533–544.

Rode KD, Atwood TC, Thiemann GW, St. Martin M, Wilson RR, Durner GM, Regehr E V., Talbot SL, Sage GK, Pagano AM, *et al.* (2020) Identifying reliable indicators of fitness in polar bears. *PLoS One* 15: 1–27.

Sciullo L, Thiemann GW, Lunn NJ (2016) Comparative assessment of metrics for monitoring the body condition of polar bears in western Hudson Bay. *J Zool* 300: 45–58.

Stevenson RD, Woods WA (2006) Condition indices for conservation: new uses for evolving tools. *Integr Comp Biol* 46: 1169–1190.

Stirling I, Thiemann GW, Richardson ES (2008) Quantitative support for a subjective fatness index for immobilized polar bears. *J Wildl Manage* 72: 568–574.

Thiemann GW, Iverson SJ, Stirling I (2006) Seasonal, sexual and anatomical variability in the adipose tissue of polar bears (Ursus maritimus). *J Zool* 269: 65–76.

Whiteman JP, Harlow HJ, Durner GM, Regehr E V., Rourke BC, Robles M, Amstrup SC, Ben-David M (2017) Polar bears experience skeletal muscle atrophy in response to food deprivation and reduced activity in winter and summer. *Conserv Physiol* 5: 1–15.
